# Celastrol mediates autophagy and apoptosis via the ROS/JNK and Akt/mTOR signaling pathways in glioma cells

**DOI:** 10.1186/s13046-019-1173-4

**Published:** 2019-05-03

**Authors:** Xihong Liu, Peiyuan Zhao, Xiujuan Wang, Lei Wang, Yingjun Zhu, Yadi Song, Wei Gao

**Affiliations:** 1 School of Traditional Chinese Medicine, Capital Medical University, Beijing, China; 2 Beijing Key Lab of TCM Collateral Disease Theory Research, Capital Medical University, Beijing, China; 3 Advanced Innovation Center for Human Brain Protection, Capital Medical University, Beijing, China; 4 Basic Discipline of Integrated Chinese and Western Medicine, Henan University of Chinese Medicine, Zhengzhou, Henan China

**Keywords:** Celastrol, Apoptosis, Autophagosome, Glioma, Cell cycle

## Abstract

**Background:**

Celastrol, a triterpene compound derived from the traditional Chinese medicine *Tripterygium wilfordii*, has been reported to possess potential antitumor activity towards various malignancies. However, the effect of celastrol on glioma cells and the underlying molecular mechanisms remain elusive.

**Methods:**

Glioma cells, including the U251, U87-MG and C6 cell lines and an animal model were used. The effects of celastrol on cells were evaluated by flow cytometry, confocal microscopy, reactive oxygen species production assay and immunoblotting after treatment of celastrol. Fisher’s exact test, a one-way ANOVA and the Mann-Whitney U-test were used to compare differences between groups. All data were analyzed using SPSS version 21.0 software.

**Results:**

Here, we found that exposure to celastrol induced G2/M phase arrest and apoptosis. Celastrol increased the formation of autophagosomes, accumulation of LC3B and the expression of p62 protein. Celastrol-treated glioma cells exhibited decreased cell viability after the use of autophagy inhibitors. Additionally, autophagy and apoptosis caused by celastrol in glioma cells inhibited each other. Furthermore, celastrol induced JNK activation and ROS production and inhibited the activities of Akt and mTOR kinases. JNK and ROS inhibitors significantly attenuated celastrol-trigged apoptosis and autophagy, while Akt and mTOR inhibitors had opposite effects.

**Conclusions:**

In conclusion, our study revealed that celastrol caused G2/M phase arrest and trigged apoptosis and autophagy by activating ROS/JNK signaling and blocking the Akt/mTOR signaling pathway.

**Electronic supplementary material:**

The online version of this article (10.1186/s13046-019-1173-4) contains supplementary material, which is available to authorized users.

## Background

Gliomas are the most common, highly proliferative and invasive primary intracranial tumors [1]. Currently, temozolomide (TMZ) is the first-line chemotherapeutic drug for glioma treatment with a definite curative effect. However, the efficacy of TMZ is frequently limited by the durability of the chemoresistance response. Therefore, research that facilitates the development of innovative drugs is urgently needed.

Celastrol is a predominantly active natural product extracted from the root bark of TCM *Tripterygium wilfordii* and it has various biological properties, such as anti-tumor, immunosuppression and weight loss activities [2–5]. In 2007, celastrol, along with artemisinin, triptolide, capsaicin, and curcumin, was reported to be a molecule that was most likely to be developed into a modern drug [6]. Previous research has shown that celastrol exhibits potential cytotoxicity in multiple tumor cells. Xu et al. reported that celastrol could inhibit the growth of ovarian cancer cells by inducing apoptosis via increased intracellular ROS accumulation in vitro and in vivo [7]. In non-small-cell lung cancer, celastrol inhibited cell proliferation and induced apoptosis through the degradation of the cancerous inhibitor of protein phosphatase 2A [8]. As a potent low-molecular-weight inhibitor, celastrol inhibited the proliferation of AML cells in vitro and prolonged the survival of mice in an in vivo model of AML [9]. Studies have shown that celastrol can inhibit the growth of glioma cells, although the detailed mechanism remains to be investigated [10, 11]. In addition, celastrol has shown neuroprotective effects in various disease models (such as Parkinson’s Disease, Alzheimer’s Disease, and Amyotrophic Lateral Sclerosis), which means that celastrol can cross the blood–brain barrier [12, 13], which may be an advantage of celastrol in the treatment of intracranial tumors.

Aberrant changes in the cell cycle commonly occur in tumor cells, and many cytotoxic agents act on cell cycle checkpoints [14]. The G2/M check point arrest is an effective mechanism adopted by many cytotoxic agents. The cyclinB1/cdc2 complex, which plays a key role in controlling the progression of the cell cycle by regulating the phosphorylation status of various proteins, is regulated by a series of proteins, including p21, Cdc25C, and Chk2 [15–17].

Studies have found that apoptosis and autophagy are two main pathways for death of tumor cells. Apoptosis is a common pattern of cell death observed with chemotherapies against all types of cancers [18]. Apoptosis is usually accompanied by typical morphological changes, including cell membrane blebbing, cell shrinkage, nuclear condensation and fragmentation, and apoptotic body formation. Autophagy, which is also known as autophagic cell death, is an evolutionarily conserved intracellular self-digestive process that maintains cellular homeostasis via lysosome-dependent machinery [19]. Beth Levine et al. demonstrated that autophagy played an extremely important role in tumor suppression [20]. Moreover, autophagy is widely recognized as a mechanism for tumor cell survival by enhancing stress tolerance and providing an alternative pathway for cancer cells to provide substantial nutrient and energy requirements [21]. Recent studies have demonstrated that a large number of antitumor drugs known to induce apoptosis also activated autophagy [22]. Therefore, further research is needed to be focused on the possible mechanism underlying celastrol-induced apoptosis or autophagy in glioma cells and determine the role of these processes and their relationship.

Reactive oxygen species (ROS) are the main molecules produced under conditions of oxidative stress, and they have long been considered to be important factors in tumorigenesis and tumor development and recurrence [23]. ROS include oxygen anions, superoxide (O_2_^−^), hydroxyl radicals and peroxides such as hydrogen peroxide (H_2_O_2_). In glioma cells, treatment with H_2_O_2_ simultaneously activated autophagy and apoptosis, which induced the membrane potential and the release of cytochrome c [24]. The generation of O_2_^−^ caused mitochondrial damage, selective degradation of mitochondria via autophagosomes and cell death of malignant glioma cells [25]. ROS can activate various signaling pathways, such as members of the MAPK family including p38, JNK and ERK1/2 [26, 27]. Activation of the JNK and p38 MAPK signaling pathways may be related to apoptosis and multiple pathophysiological processes during stress [23]. As a classic signaling pathway, the AKT/mTOR pathway has also been reported to mediate antitumor drug-induced apoptosis and autophagy [28].

In the present study, we aimed to investigate the antitumor effects and possible mechanisms underlying the impact of celastrol on glioma cells both in vitro and in vivo. We elucidated that celastrol induced G2/M-phase arrest, apoptosis, and autophagy in glioma cells by modulating the ROS/JNK and Akt/mTOR signaling pathways. In addition, we found that autophagy caused by celastrol played a role in promoting cell survival. Celastrol-induced apoptosis and autophagy inhibited each other.

## Methods

### Cells and cell culture

The human glioma cell lines U251 and U87 and rat glioma cell line C6 were purchased from the Cell Resource Center (IBMS, CAMS/PUMC, Beijing, China). The U251 cells were cultured in MEM (Corning, NY, USA), and the U87 cells were cultured in MEM-NEAA supplemented with 10% FBS, 100 U/ml penicillin and 100 μg/ml streptomycin. The C6 cells were cultured in F10 with 15% horse serum (HyClone, Logan, UT, USA) containing 2.5% FBS, 100 U/ml penicillin and 100 μg/ml streptomycin. The cells were maintained at 37 °C in a humidified incubator with 5% CO_2_. Astrocytes were prepared from primary cell cultures of neocortical tissues from one-day-old SD rats. Briefly, following the removal of the meninges, the cortices were mechanically dissociated and digested with 0.25 mg/mL trypsin, triturated in DMEM by pipetting, and filtered to remove large debris. The isolated cells were suspended in DMEM containing 10% FBS and plated on flasks coated with Poly-D-lysine. After one week, mixed glial cells were shaken for 16 h at 240 rpm. Detached cells, which consisted of microglia and oligodendrocytes, were removed. Attached cells were maintained as astrocyte-enriched cultures. After 3–5 days of additional culture, the cells were plated into appropriate plates for the experiments.

### Reagents and antibodies

Celastrol (purity of > 99%) was purchased from Pharmacodia Co., Ltd. (Beijing, China). N-Acetyl-L-cysteine (NAC), 3-methyladenine(3-MA), z-VAD-fmk (z-VAD), SP600125 (SP), MK2206 (MK) and rapamycin (Ra) were purchased from Selleckchem (Houston, TX, USA). Chloroquine diphosphate salt (CQ) and TMZ were purchased from Sigma (St. Louis, MO, USA). Antibodies against caspase-3 (cat. no. 9664), SQSTM1/p62 (cat. no. 5114), cyclin B1 (cat. no. 4138) and phospho-cdc2 (cat. no. 4539) were obtained from Cell Signaling Technology (Beverly, MA, USA). Antibodies against caspase-8 (cat. no. ab 25,901), caspase-9 (cat. no. ab32539), cleaved PARP (cat. no. ab32064), Beclin-1(cat. no. ab207612), Akt (cat. no. ab32505), phospho-Akt (cat. no. ab192623), JNK (cat. no. ab179461), phospho-JNK (cat. no. ab124956), mTOR (cat. no. ab2732), phospho-mTOR (cat. no. ab109268), p38 (cat. no. ab170099), phospho-p38 (cat. no. ab195049), Chk2 (cat. no. ab47433), phospho-Chk2 (cat. no. ab59408), Cdc25C (cat. no. ab226958), phospho-Cdc25C (cat. no. ab47322) and p21 (cat. no. ab109199) were purchased from Abcam (Cambridge, MA, UK). Antibody against LC3B (cat. no. 6008–1-Ig) was purchased from Sigma. Antibody against β-actin (cat. no. 6008–1-Ig) was purchased from Proteintech (Chicago, IL, USA). The plasmid ptfLC3 was purchased from Addgene Repository (http://www.addgene.org).

### Cell viability assay

Inhibition of glioma cell proliferation by celastrol was detected by a CCK8 assay (Dojindo, Kumamoto, Japan). Briefly, the cells were passaged in 96-well plates with a density of 2.5 × 10^3^ cells/well. After 24 h, the cells were treated with gradient concentrations of celastrol (0.3, 1, 3 and 10 μM) for various durations (12, 24, 36 and 48 h). After incubation, the cells were treated with a mixture of CCK8 and MEM and incubated for 0.5–1 h. The absorbance at 450 nm was determined by a microplate reader (Molecular Devices, Sunnyvale, CA, USA).

### Clone formation assay

Cells were cultured in 6-well plates with a density of 100–400 cells/well. After 24 h, the cells were treated with various concentrations of celastrol (0.03, 0.1, 0.3 and 1 μM) for approximately 10 days or treated with celastrol for 24 h and left untreated for approximately 10 days to allow for the generation of colonies. Then, the medium was discarded, and the cells were washed with PBS 3 times. After being fixed with 4% paraformaldehyde, the colonies were stained with 0.1% crystal violet for 15 mins. The clones with more than 50 cells were counted. The images were captured by a digital camera.

### Cell cycle analysis by flow cytometry

The role of celastrol in cell cycle distribution was monitored by flow cytometry with PI/RNase staining buffer (BD Biosciences, San Jose, CA, USA). Briefly, cells were passaged in 6-well plates at a density of 3 × 10^5^ cells/well. After 24 h, the cells were exposed to various concentrations of celastrol (0, 0.3, 1, 3, and 10 μM) for 24 h. Then, the cells were harvested, fixed with 75% ethanol at -20 °C overnight, and stained with PI/RNase staining buffer for 15 mins. The cell cycle analyses were performed with a NovoCyte instrument (ACEA Biosciences, San Diego, CA, USA.), and the data were analyzed by using NovoExpress software (ACEA).

### Morphological changes due to apoptosis

Characteristic morphological changes associated with apoptosis were assessed by fluorescence microscopy using Hoechst 33342 staining. Briefly, cells were cultured at a density of 3 × 10^4^/ml in 96-well plates and then treated with celastrol for 24 h. Then, the cells were fixed with 4% paraformaldehyde for 15 mins and stained with Hoechst 33342 solution for 30 mins at room temperature in the dark. Nuclear fragmentation and chromatin condensation were observed with a fluorescence microscope (Olympus, Tokyo, Japan) after the cells were washed 3 times with PBS.

### Flow cytometric analysis of apoptosis

Apoptosis was assessed by a FITC Annexin V Apoptosis Detection Kit I (BD Biosciences, San Diego, CA, USA) according to the provided protocol. The samples were analyzed by a flow cytometer (LSRFortessa SORP, BD, San Jose, CA, USA).

### Measurement of mitochondrial membrane potential

Mitochondrial membrane potential (MMP) was measured with a MMP Assay Kit with JC-1 (Beyotime, Jiangsu, China) according to the manufacturer’s instructions. Briefly, cells were seeded in six-well plates at a density of 2.5 × 10^5^/ml and then treated with celastrol (0–10 μM) for 24 h. The cells were then harvested and resuspended in 500 μl of MEM. Then, 500 μl of JC-1 staining solution was added, and the cells were incubated for 20 mins at 37 °C in a CO_2_ incubator. The results were analyzed by flow cytometry, and mitochondrial depolarization was evaluated by measuring the decrease in the red/green fluorescence intensity ratio.

### mRFP-EGFP-LC3 puncta assay

U251 cells were transiently transfected with ptfLC3 (mRFP-EGFP-LC3) plasmid to examine the formation of fluorescent puncta of autophagosomes. Cells were cultured in 24-well plates and transfected with 0.8 μg/well mRFP-EGFP-LC3 plasmid using Lipofectamine 2000 (Invitrogen, Carlsbad, CA, USA). After transfection, the cells were treated with or without 1.5 μM celastrol for 24 h and then incubated with DAPI for 15 mins. Image acquisition was performed using a Leica confocal laser scanning microscope.

### Measurement of intracellular ROS generation

Intracellular ROS production was detected using the ROS Assay Kit (Beyotime). Cells were plated in six-well plates at a density of 2.5 × 10^5^/ml and treated with celastrol in the absence or presence of NAC (a ROS scavenger) and SP (a JNK inhibitor). The cells were then incubated with 10 μM DCFH-DA at 37 °C for 30 mins. Then, then cells were washed with serum-free MEM, and the ROS levels were determined by fluorescence microscopy (Leica, Wetzlar, Germany) and flow cytometry (BD Biosciences, San Jose, CA, USA).

### Western blotting analysis

The glioma cells or tissues were lysed with cool RIPA lysis buffer. Proteins were extracted following the standard protocol and the concentration was measured using a BCA protein assay kit. Equal amounts of protein (30 μg) were separated by 10–15% SDS-PAGE and electrotransferred to PVDF membranes. The membranes were blocked with 5% skim milk for 1 h and then incubated overnight at 4 °C with the following antibodies: β-action (1:50000), LC3B (1:10000), SQSTM1/p62 (1:3000), (Beclin1 (1:4000), Akt (1:10000), phospho-Akt (p-Akt, 1:5000), p38 (1:5000), phospho-p38 (p-p38, 1:1500), JNK (1:3000), phospho-JNK (p-JNK, 1:3000), mTOR (1:5000), phospho-mTOR (p-mTOR, 1:30000), caspase-8 (1:3000), caspase-9 (1:3000), cleaved PARP (1:3000), cleaved caspase-3 (1:3000), Chk2 (1:1000), phospho-Chk2 (p-Chk2,1:500), Cdc25C (1:1000), phospho-Cdc25C (p-Cdc25C, 1:1000), cyclin B1 (1:1500), phospho-cdc2 (p-cdc2, 1:2000), p21 (1:1500). Membranes were then incubated with HRP-conjugated goat anti-mouse or anti-rabbit IgG antibodies (1:5000) for 1 h at 37 °C, detected by an enhanced chemiluminescent detection system and quantified using ImageJ.

### Human glioma xenograft experiment

All animal procedures were performed according to the guidelines of the Animal Experiments and Experimental Animal Welfare Committee of Capital Medical University (Approval number: AEEI-2017-119). Healthy male BALB/c-nu mice (6-8 weeks old, 18-20 g) were purchased from Beijing Vital River Laboratory Animal Technology Co., Ltd. The mice were kept in standard cages in a room with a controlled environment in terms of temperature (25 ± 2 °C), humidity (40-50%) and light (12 h light/dark cycle), with commercial standard solid rodent chow and water provided ad libitum in the Experimental Animal Center of Capital Medical University. Approximately 3.5 × 10^5^ U251 cells (> 95% viability) in a volume of 5 μl were stereotactically injected into the right striatum (2 mm lateral, 1 mm anterior to the bregma, and 3 mm deep) by using a small animal stereotactic frame (RWD Life Science, Shenzhen, China).

### TUNEL assay

The apoptotic response of tumor tissues was identified using a TUNEL assay with an In Situ Cell Death Detection Kit, Fluorescein (Roche Diagnostics, Mannheim, Germany), according to the manufacturer’s instructions.

### Histopathology and immunohistochemistry

Formalin-fixed tissue samples were embedded in paraffin, and 4-μm sections were cut. Primary tumor, heart, liver, spleen, lung, and kidney sections were stained with hematoxylin and eosin (H&E) for routine histological examinations and morphometric analysis. Brain slices were immunostained with Ki67 (1:400), phospho-JNK (1:100), and cleaved caspase-3 (1:100). Images were captured using a Leica microscope.

### Statistical analysis

Quantitative data were presented as the mean ± SD from at least three independent experiments. Fisher’s exact test, one-way ANOVA and the Mann-Whitney U-test were used to compare differences between groups. All data were analyzed using SPSS version 21.0 software (IBM Corporation, Chicago, USA). Statistical significance was indicated as *P* < 0.05.

## Results

### Celastrol inhibits the proliferation of glioma cells and exhibits low cytotoxicity towards astrocytes

**Fig. 1:**
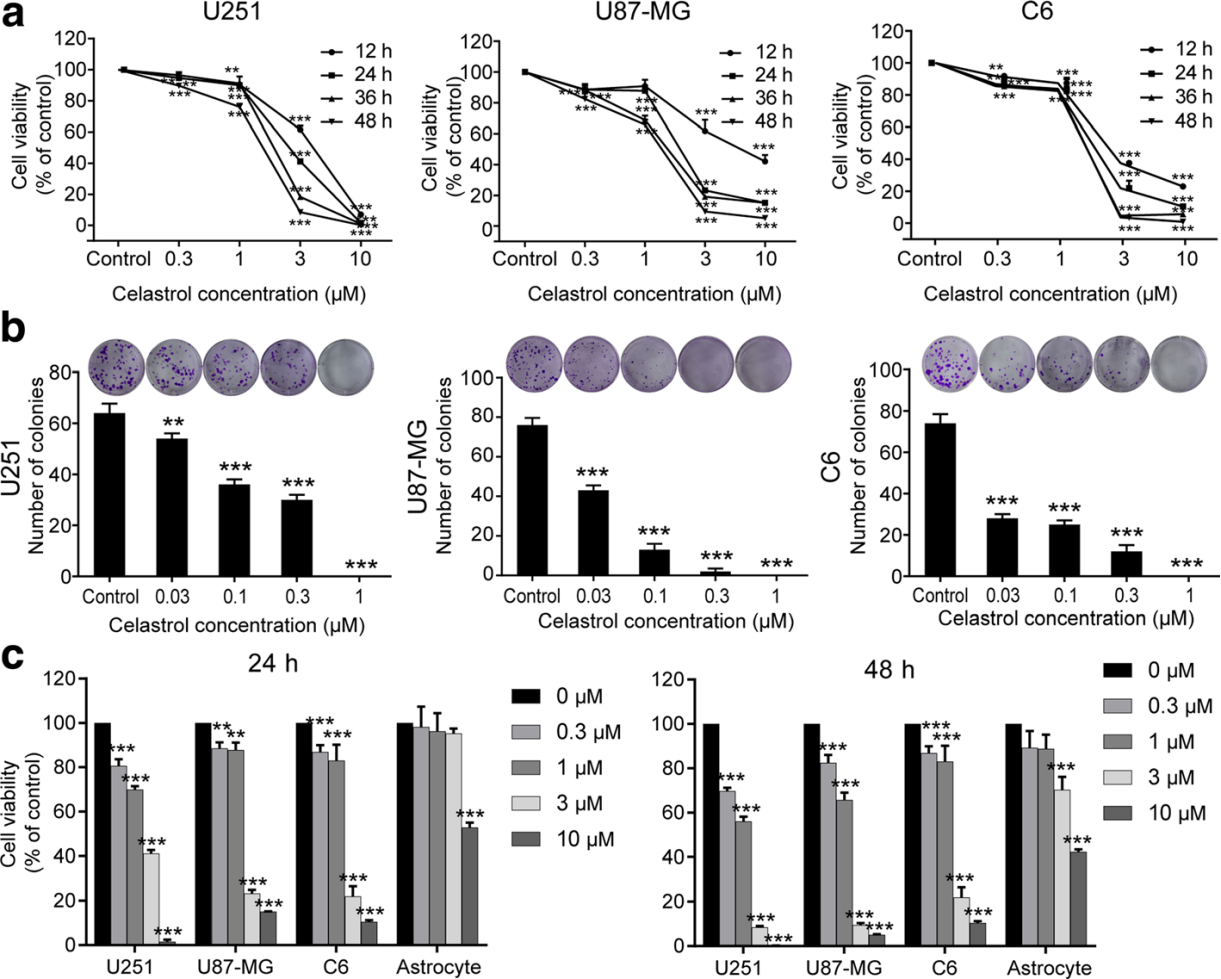
Celastrol (Cel) inhibited cell viability in glioma cells. **a** Three glioma cell lines (U251, U87-MG and C6) were treated with celastrol (0, 0.3, 1, 3 and 10 μM) for 12, 24, 36 and 48 h. Cell viability was measured by CCK8 assay. **b** U251, U87-MG and C6 cells were treated with celastrol (0, 0.03, 0.1, 0.3 and 1 μM) for approximately 10 days. Cell colony formation was evaluated by a clone-formation assay. **c** Comparison of the effect of celastrol on three glioma cell samples with that on the astrocytes for 24 and 48 h. Three glioma cell lines and astrocyte cells were treated with celastrol (0, 0.3, 1, 3 and 10 μM) for 24 and 48 h. Cell viability was measured by CCK8 assay. Data are presented as the Mean ± SD (*n* = 3). **P < 0.01, ***P < 0.001, significantly different compared with the untreated control group

To investigate the antiproliferative activity and cytotoxicity of celastrol on glioma cells, U251, U87-MG and C6 cells were treated with various concentrations of celastrol for 12, 24, 36 and 48 h, and cell viability and morphology were measured by a CCK8 assay and light microscopy, respectively. We found that celastrol inhibited the growth and induced morphological alterations in the glioma cells in a time- and dose-dependent manner (Fig. 1a and Additional file 1: Figure S1a). When cells were treated with 10 μM celastrol for 24 or 48 h, almost all U251 cells were dead. The survival rates of U87-MG and C6 were approximately 10, 15% at 24 h and 5, 0.8% at 48 h, respectively. The IC_50_ values of celastrol were 1.62 μM (24 h) and 0.88 μM (48 h) for U251 cells, 1.96 μM (24 h) and 1.24 μM (48 h) for U87-MG cells, and 1.61 μM (24 h) and 1.06 μM (48 h) for C6 cells. To reflect the two important traits of population dependence and proliferative capacity in treated cells, we carried out a colony formation experiment. Cells were plated into culture plates at a very low density (200 cells/well) and treated with lower various concentrations of celastrol (0, 0.03, 0.1, 0.3 and 1 μM). The colony formation assay confirmed that the celastrol treatment significantly decreased the number of colonies (Fig. 1b and Additional file 1: Figure S1b). Interestingly, celastrol exhibited a reduced inhibitory effect on primary cultured astrocyte cells. As shown in Fig. 1c and Additional file 1: Figure S1c, when treated with 3 μM celastrol, the activity of the three glioma cell lines was significantly inhibited whereas the activity of the primary cultured astrocytes was less affected. The IC_50_ values for astrocyte cells were 12.54 μM (24 h) and 7.22 μM (48 h). These results demonstrated that celastrol exhibits antiproliferative activity towards glioma cells and has low cytotoxicity against normal glial cells.

### Celastrol induces cell cycle phase arrest in glioma cells

**Fig. 2:**
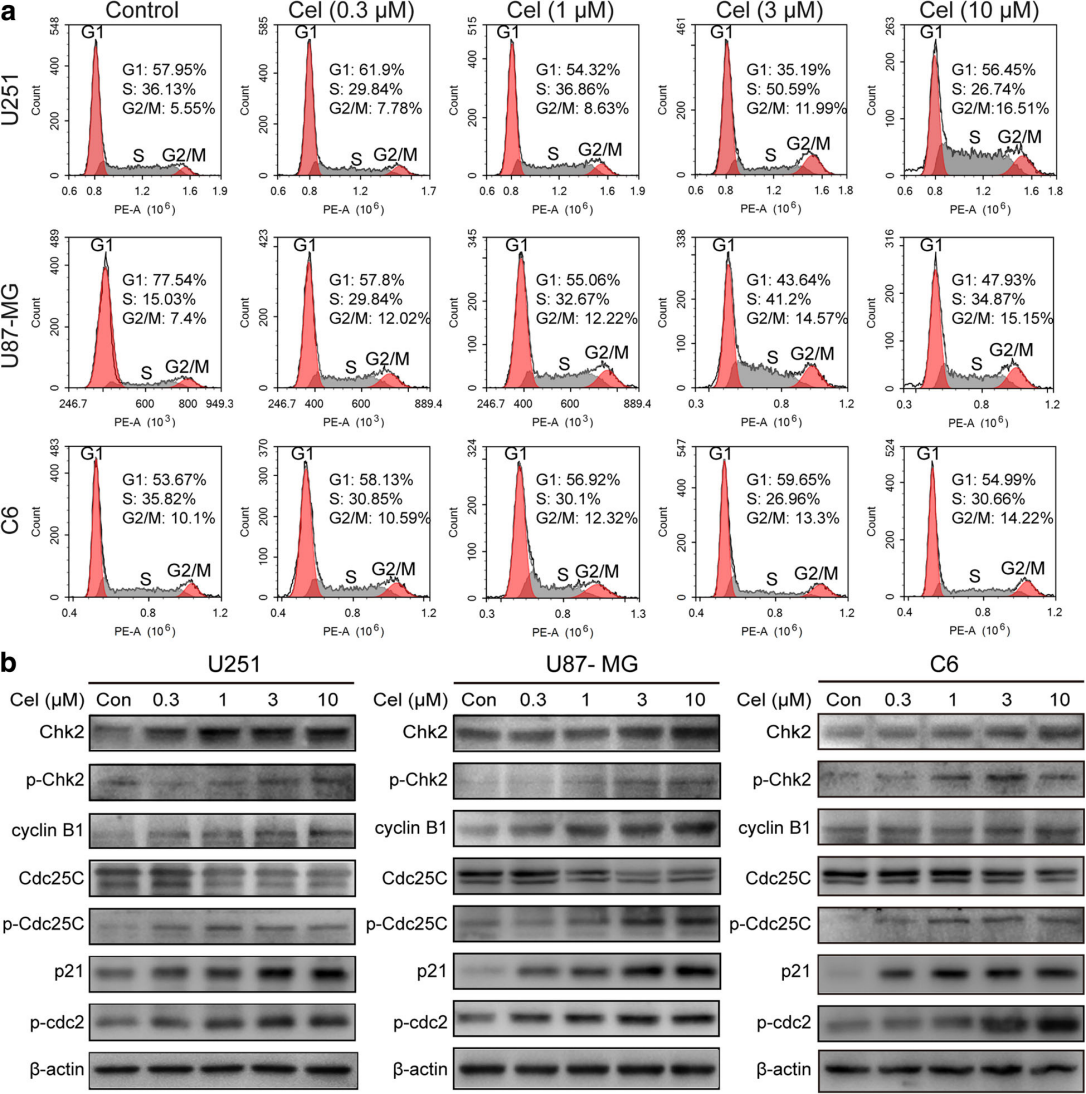
Celastrol induced G2/M cell cycle arrest in glioma cells. **a** Cells were treated with celastrol (0, 0.3, 1, 3 and 10 μM) for 24 h and cell cycle distribution was analyzed by flow cytometry. **b** U251, U87-MG and C6 cells were treated with celastrol (0, 0.3, 1, 3 and 10 μM) for 24 h. The levels of cell cycle-associated protein expressions were analyzed by western blotting. β-actin was used as an internal control

To verify whether celastrol inhibits the growth of glioma cells by regulating the cell cycle, we tested the cell cycle distribution in cells treated with various concentrations of celastrol for 24 h. As shown in Fig. 2a and Additional file 2: Figure S2, celastrol increased the percentage of the G2/M phase in the cell cycle, although the changes in cell cycle were not exactly the equivalent in the U251, U87-MG and C6 cells. These changes were accompanied by a decrease in the G1 phase and an increase in the proportion of the S phase in U87-MG cells in a dose-dependent manner. Furthermore, the expression of cell-cycle-regulated proteins was measured by western blotting. The results showed that celastrol upregulated the expression of Chk2, p-Chk2, cyclin B1, p-Cdc25C, p-cdc2 and p21 and downregulated the expression of Cdc25C (Fig. 2b). The aforementioned results suggested that celastrol induced cell cycle arrest by regulating cell-cycle-related proteins.

### Celastrol induces apoptosis in glioma cells

**Fig. 3:**
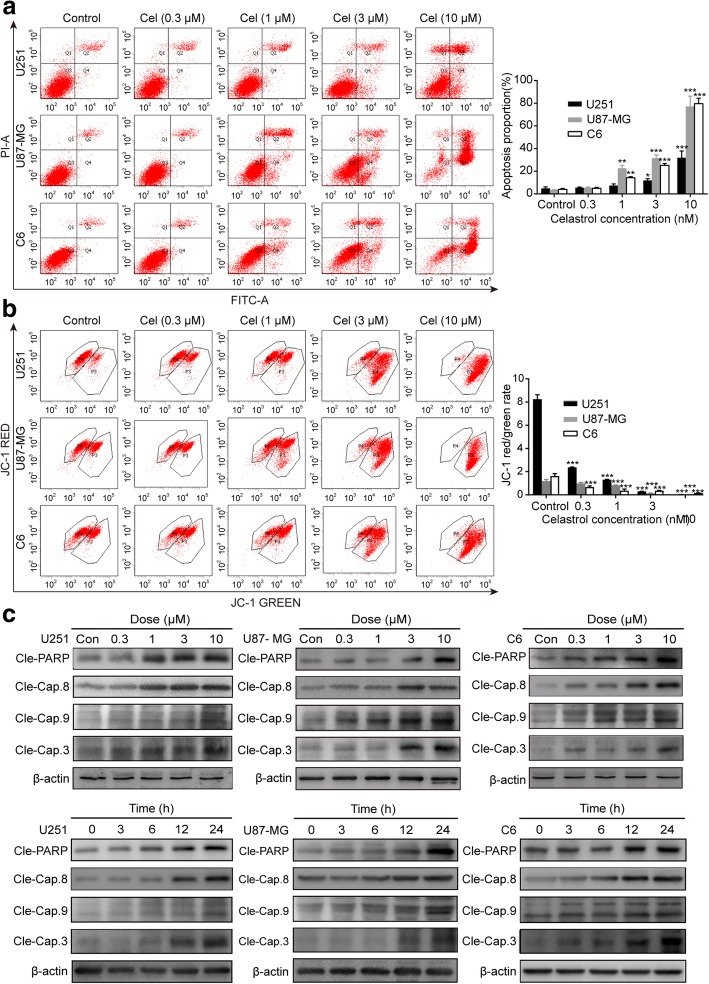
Celastrol induced intrinsic and extrinsic apoptosis in glioma cells. **a** Cells were treated with celastrol (3 μM) for 24 h, early and late apoptotic cells were analyzed using Annexin V-FITC/PI flow cytometry. The histograms indicate the proportion of apoptosis from three separate experiments. **b** The MMP was measured with the fluorescent mitochondrial probe JC-1 and assessed by flow cytometry. The histograms indicate the red/green fluorescence intensity ratio. **c** Cells were treated with various concentrations of celastrol for 24 h or incubated with celastrol (3 μM) for different durations. The apoptosis-related proteins cleaved PARP and Caspase-3, -8, -9 were analyzed by western blotting. β-actin was used as an internal control. Data are presented as the Mean ± SD (*n* = 3). *P < 0.05, **P < 0.01, ***P < 0.001, significantly different compared with the untreated control group

**Fig. 4:**
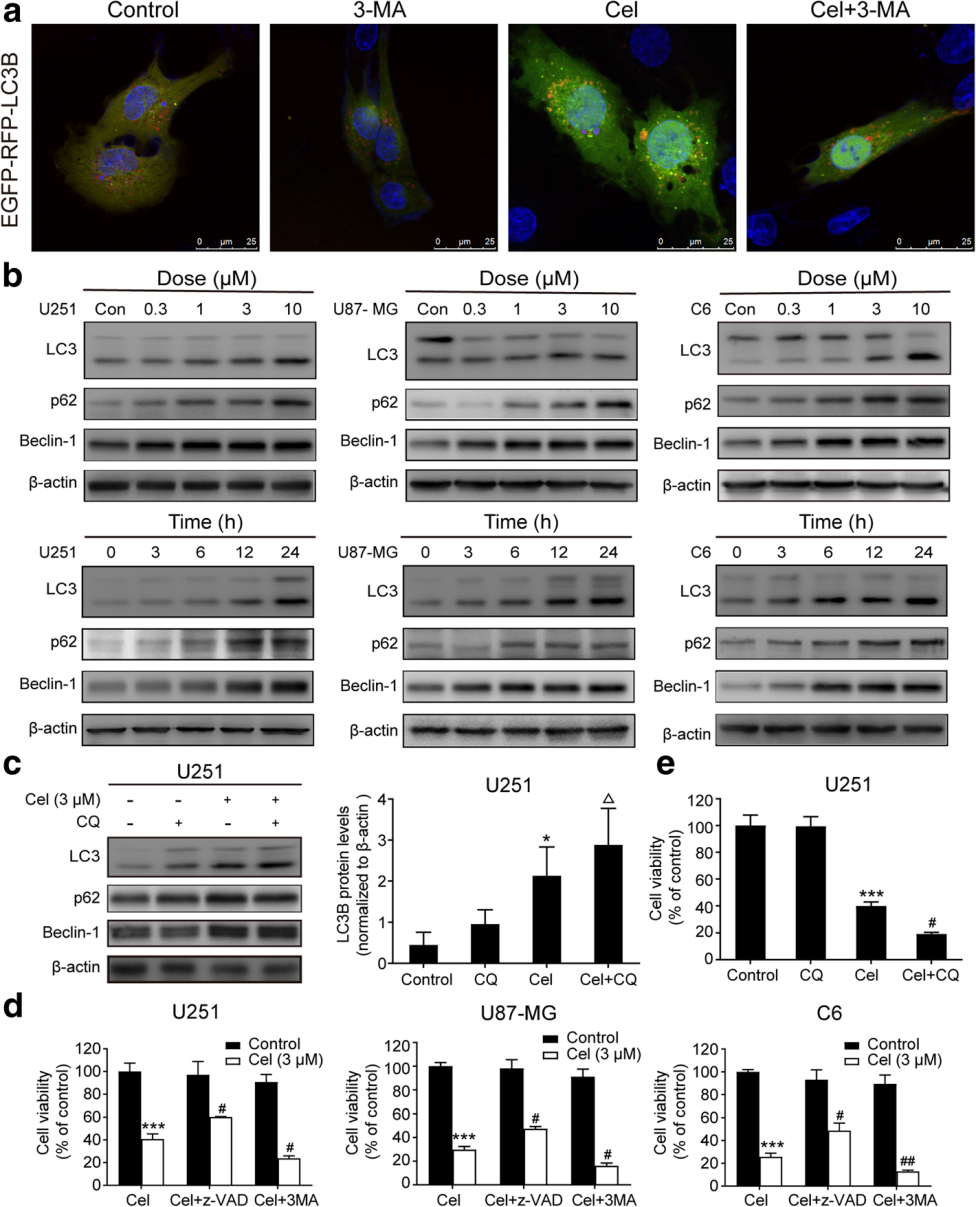
Celastrol triggered autophagy in glioma cells, and inhibition of autophagy increased celastrol-induced cell death. **a** U251 cells were transiently transfected with the mRFP-EGFP-LC3 plasmid for 24 h and then treated with or without celastrol (1.5 μM) for 24 h. The images were captured using a confocal microscope. Scale bars = 25 μm. **b** Cells were treated with various concentrations of celastrol for 24 h or incubated with celastrol (3 μM) for different durations. The autophagy-related proteins LC3, P62 and Beclin-1 were detected by western blotting. **c** CQ (25 μM) was added to cells 2 h before celastrol treatment. Then, cells were treated with celastrol for 24 h. The autophagy-related proteins LC3, P62 and Beclin-1 were detected by western blotting. **d** z-VAD (30 μM) or 3-MA (3 mM) was added to cells 2 h before celastrol treatment. After 24 h, cell viability was determined. **e** CQ was added to cells 2 h before celastrol treatment. Then, cells treated with celastrol for 24 h. Cell viability was determined by CCK8. β-actin was used as an internal control. Data are presented as the Mean ± SD (*n* = 3). *P < 0.05, ***P < 0.001, significantly different compared with the untreated control group. ^#^*P < 0.05*, ^##^*P < 0.01*, significantly different compared with the celastrol treatment group. ^△^*P < 0.05*, significantly different compared with the CQ treatment group

We further explored whether celastrol could inhibit cell proliferation by inducing apoptosis in glioma cells. Hoechst 33342 was used to examine the morphologic changes in cells treated with celastrol. The results showed the occurrence of different degrees of cell shrinkage, chromatin condensation, and nuclear fragmentation after treatment with celastrol for 24 h (Additional file 3: Figure S3). Furthermore, we analyzed the apoptosis by flow cytometry using FITC Annexin V/PI double staining. As shown in Fig. 3a, both early and late apoptotic cells were all increased in a dose-dependent manner in cells exposed to celastrol. Next, MMP was measured by flow cytometry using the fluorescent mitochondrial probe JC-1. We found that a considerable amount of the red fluorescence was converted to green, indicating that MMP was diminished following celastrol treatment (Fig. 3b). Then, we tested the expression of the central signaling proteins related to apoptosis by western blotting. As shown in Fig. 3c, celastrol remarkedly increased the protein levels of cleaved PARP and caspase-3, -8, and -9 in a time and dose-dependent manner. In addition, we used broad-spectrum caspase inhibitor z-VAD to block celastrol-induced apoptosis in glioma cells. Consistently, the administration of z-VAD prevented a portion of cell death induced by celastrol (Fig. 4d). The aforementioned results demonstrated that celastrol provokes apoptosis by activating both extrinsic and intrinsic pathways.

### Celastrol triggers autophagy, which promotes cell survival

We then investigated whether celastrol mediates autophagy in glioma cells. MRFP-EGFP-LC3 plasmids were transiently transfected to glioma cells to analyze the formation of fluorescent puncta of autophagosomes. Upon exposure to celastrol for 24 h, LC3 puncta formation, which marks the occurrence of autophagy in U251 cells, exhibited a significant increase. This change disappeared when we used the autophagy inhibitor 3-MA to block the beginning of autophagy (Fig. 4a and Additional file 4: Figure S4a). In addition, the expression of autophagy-related proteins was tested by western blotting. We found that celastrol treatment markedly improved the expression of LC3B and Beclin-1 in both time- and concentration-dependent manners. Interestingly, the level of p62 also increased (Fig. 4b and Additional file 4: Figure S4b). To further investigate the molecular mechanism of autophagy, we used the autophagy inhibitor CQ, which increases the pH of the lysosome to limit the degradation of the autophagosome. We tested the autophagy-related proteins by western blotting, and the results indicated that the accumulation of LC3B was not obviously different after treatment with the combination of celastrol + CQ compared with that observed after treatment with celastrol alone. However, the levels of LC3B were higher with celastrol + CQ than with CQ alone (Fig. 4c). These results suggested that celastrol can induce the formation of autophagosomes accompanied by the inhibition of lysosomal degradation function in glioma cells in both concentration- and time-dependent manners.

To clarify the role of celastrol-triggered autophagy, the autophagy inhibitors 3-MA and CQ were used to pretreat the cells before the celastrol treatment to block celastrol-induced autophagy in glioma cells. Then, we analyzed the cell viability in the presence of 3-MA and CQ. As shown in Fig. 4d and e, when autophagy was inhibited, cell viability was decreased compared with that of celastrol treatment alone. The results suggested that the autophagy mediated by celastrol promotes survival.

### Inhibition of celastrol-induced autophagy promoted apoptosis, and suppression of caspase-dependent apoptosis strengthened autophagy

**Fig. 5:**
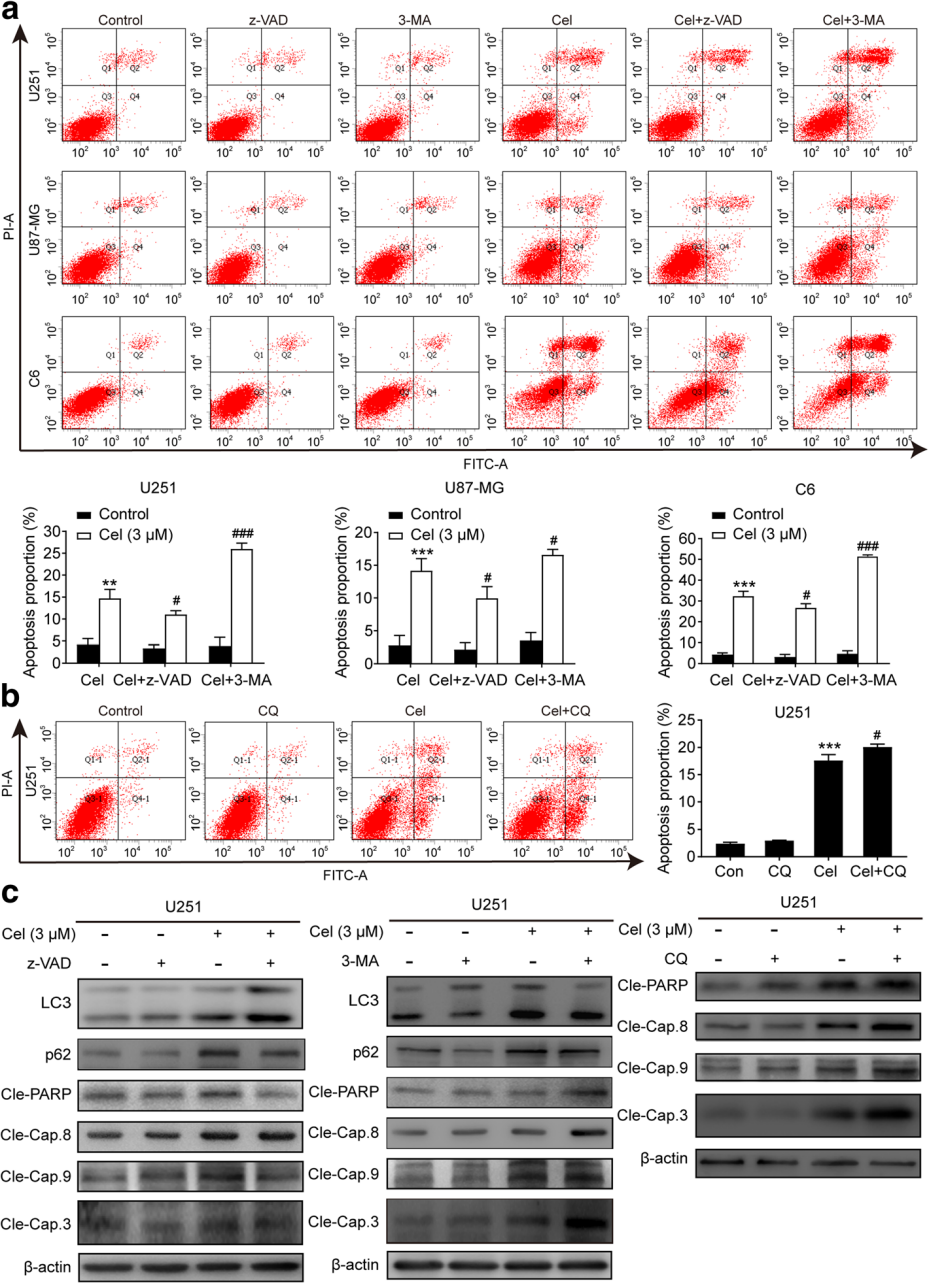
Inhibition of autophagy promoted apoptosis induced by celastrol and repressed caspase-dependent apoptosis increased the expression of autophagy-related proteins. **a** Cells were preincubated with z-VAD or 3-MA, and then treated with celastrol for 24 h. Early and late apoptotic cells were analyzed using Annexin V-FITC/PI flow cytometry. The histograms indicate the proportion of cells undergoing apoptosis from three separate experiments. **b U**251 cells were preincubated with CQ for 2 h, and then treated with celastrol for 24 h. Early and late apoptotic cells were analyzed using Annexin V-FITC/PI flow cytometry. The histograms indicate the proportion of cells undergoing apoptosis from three separate experiments. **c** U251 cells were preincubated with z-VAD or 3-MA or CQ, and then treated with celastrol for 24 h. The expression levels of LC3, P62, cleaved PARP and Caspase-3, -8, -9 were detected by western blotting. β-actin was used as an internal control. Data are presented as the Mean ± SD (n = 3). **P < 0.01, ***P < 0.001, significantly different compared with the untreated control group. ^*#*^*P* < 0.05, ^*###*^*P* < 0.001, significantly different compared with the celastrol treatment group

We then probed the interation between these two processes. After inhibiting the initiation of autophagy with 3-MA and limiting the degradation of the autophagosome with CQ, the apoptotic cells were measured by flow cytometry. It was shown in Fig. 5a and b that early and late apoptotic cells were all significantly enhanced in the 3-MA or CQ and celastrol combined treatment compared with the celastrol or 3-MA or CQ treatments alone. When 3-MA or CQ was used alone, significant changes in cell apoptosis were not observed compared with the control group. A western blotting assay was used to detect the expression of key proteins in autophagy and apoptosis. As shown in Fig. 5b and Additional file 5: Figure S5, when 3-MA or CQ was used alone, obvious changes were not observed in the expression of the apoptosis-related proteins cleaved PARP and cleaved caspase-3, -8 and -9. However, 3-MA or CQ could promote the expression of apoptosis-related proteins in the presence of celastrol compared with the control and celastrol groups. Similarly, suppression of the caspase-family proteins associated with apoptosis via z-VAD enhanced the level of the autophagy marker protein LC3B and inhibited the level of p62 (Fig. 5c and Additional file 5: Figure S5). These results indicate an inhibitory effect of celastrol on autophagy and apoptosis of glioma cells and vice versa.

### Celastrol induces ROS generation, JNK activation and blocks the Akt/mTOR signaling pathway in glioma cells

**Fig. 6:**
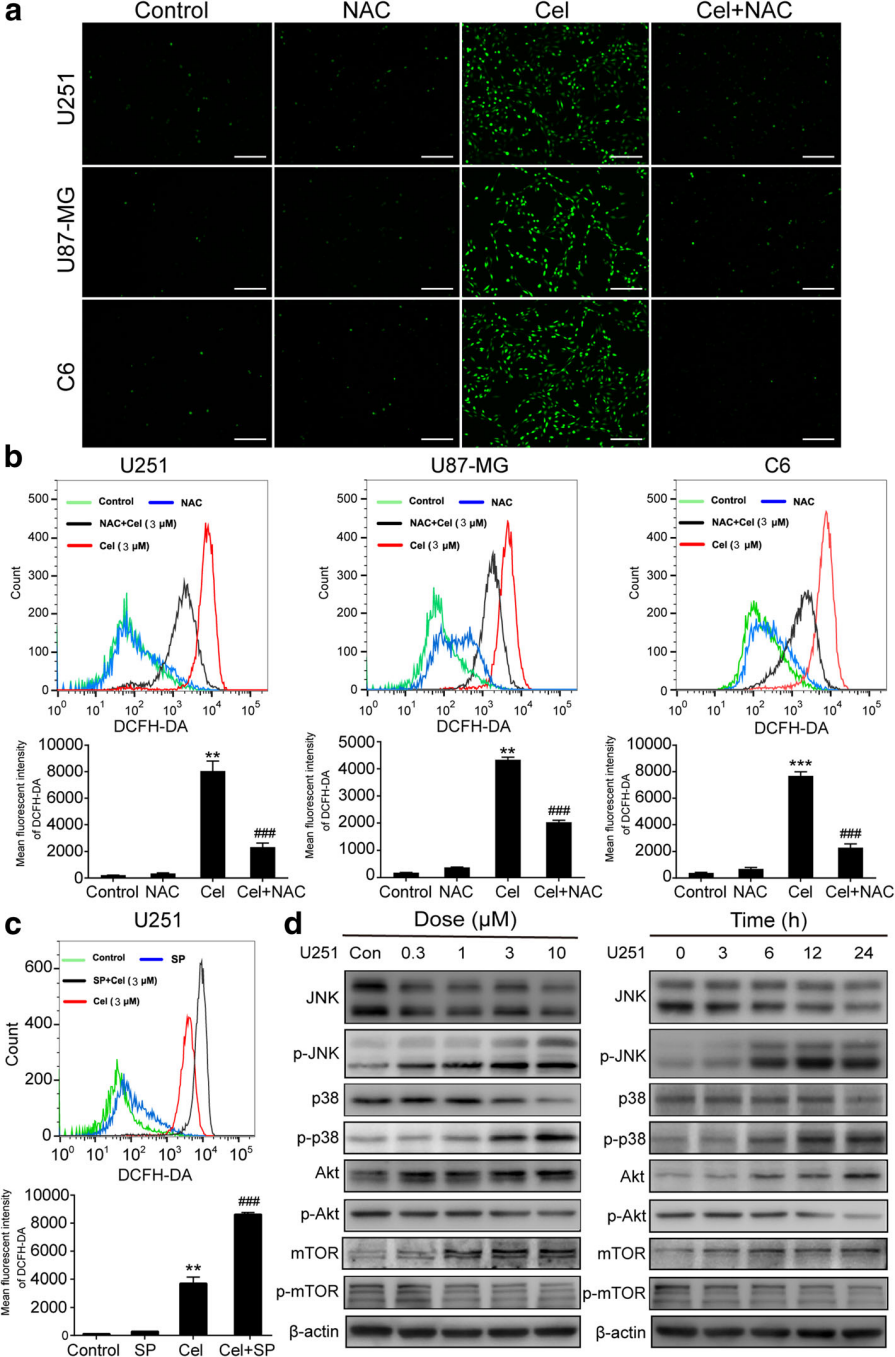
Celastrol induced ROS generation, phosphorylation of JNK and interrupted the Akt/mTOR signaling pathway in glioma cells. **a, b and c** Glioma cells were treated with 3 μM celastrol in the presence or absence of NAC (5 mM) or SP (40 μM) for 12 h. Cells were stained with 10 μM DCFH-DA at 37 °C in the dark for 20 mins, and the ROS level was determined by fluorescence microscopy and flow cytometry. Quantitative analysis of ROS generation was shown in histograms. Scale bars = 200 μm. **d** U251 cells were treated with various concentrations of celastrol for 24 h or incubated with celastrol (3 μM) for different durations. The expression levels of p-JNK, JNK, p-p38, p38, p-Akt, Akt, p-mTOR and mTOR were detected by western blotting. β-actin was used as an internal control. Data are presented as the Mean ± SD (n = 3). **P < 0.01, ***P < 0.001, versus control, ^*###*^*P <* 0.001, versus celastrol treatment group

Mitochondria are the main cellular sites for ROS production, which promotes sustained JNK activation [29, 30]. The changes in MMP were measured by flow cytometry (Fig. 3b). Therefore, we investigated the generation of ROS in U251 cells treated with celastrol. The generation of ROS was measured using the fluorescent probe DCFH-DA by fluorescence microscopy and flow cytometry. As shown in Fig. 6a, a markly enhanced fluorescent signal was observed in cells treated with celastrol compared to the signal in untreated cells. When we used the ROS scavenger NAC, this change was diminished. The flow cytometry results suggested that the mean fluorescent intensity of DCFH-DA was significantly increased in cells treated with celastrol, which can be scavenged by NAC (Fig. 6b). The aforementioned results suggest that celastrol induces ROS generation.

**Fig. 7:**
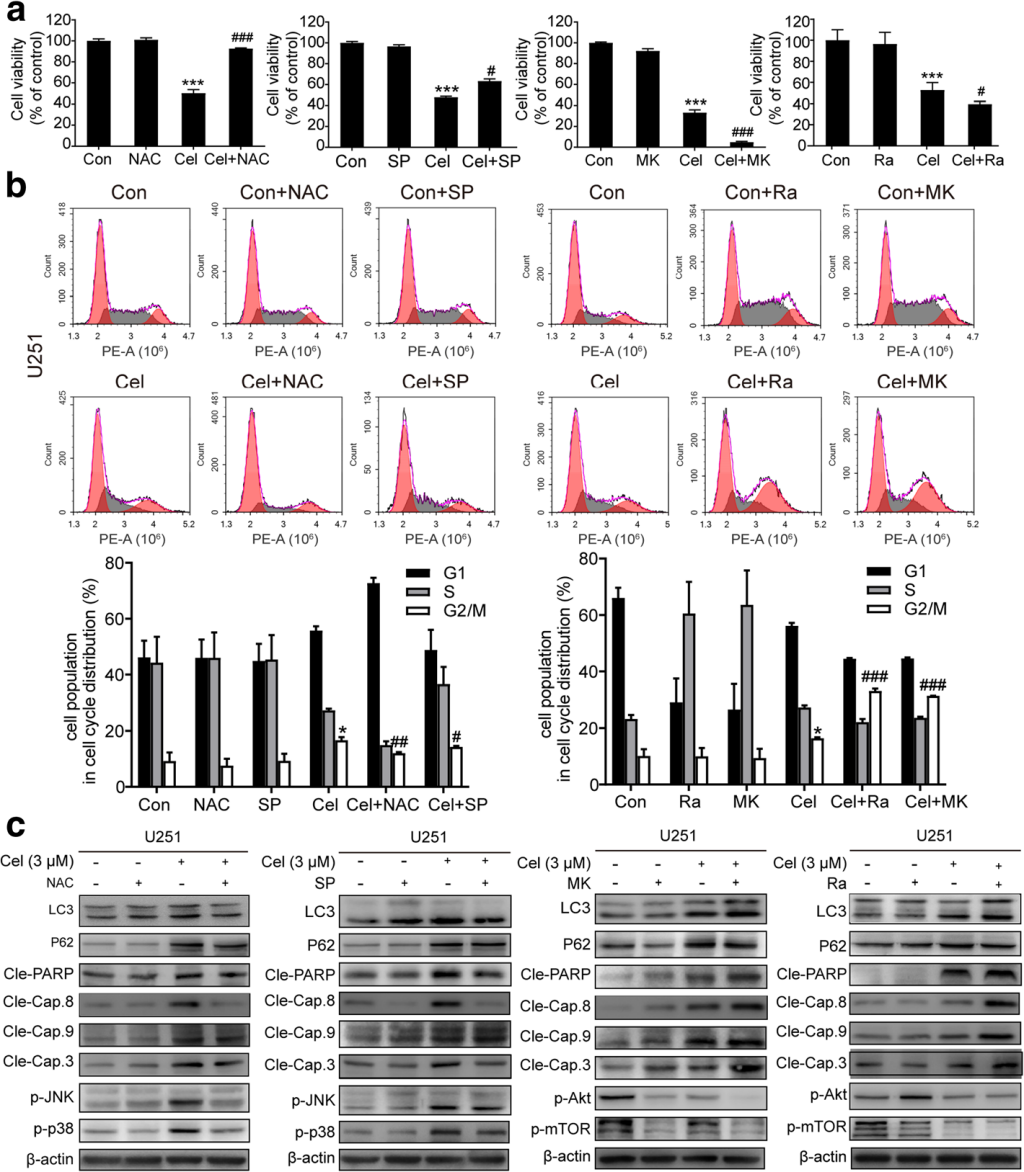
Roles of ROS/JNK and Akt/mTOR signaling pathway in G2/M phase arrest, apoptosis and autophagy triggered by celastrol. Cells were pretreated with MK (2 μM) for 5 h or Ra (1 μM) or NAC or SP for 2 h, and then treated with 3 μM celastrol for 24 h. **a** Cell viability was measured by CCK8 assay. **b** Cell cycle distribution was evaluated by flow cytometry. The cell cycle distribution percentages are presented as histograms. **c** The apoptosis-related proteins, cleaved PARP and Caspase-3, -8, -9 and the autophagy-related proteins, LC3 and P62 as well as p-JNK, p-p38 were analyzed by western blotting. β-actin was used as an internal control. Data are presented as the Mean ± SD (*n* = 3). *P < 0.05, ***P < 0.001 significantly different compared with the control group, ^*#*^*P* < 0.05, ^*##*^*P* < 0.01, ^*###*^*P* < 0.001 significantly different compared with the celastrol treatment group

Next, we tested whether ROS are the upstream signal molecules of JNK in cells treated with celastrol, and we examined the effect of celastrol on the JNK and Akt/mTOR signaling pathways. Surprisingly, the flow cytometry results suggested that SP, a JNK inhibitor, can increase ROS generation upon treatment with celastrol (Fig. 6c). When ROS generation was inhibited by NAC, the phosphorylation level of JNK is clearly reduced (Fig. 7c). In addition, western blotting analysis showed that celastrol increased the phosphorylation of p38 and JNK and decreased the phosphorylation of Akt and mTOR in both concentration- and time-dependent manners (Fig. 6d). In addition, MK (an Akt inhibitor) notably inhibited Akt and mTOR phosphorylation, while Ra (a mTOR inhibitor) did not suppress Akt phosphorylation (Fig. 7c). Collectively, these results suggested that celastrol activated ROS/JNK signaling and blocked the Akt/mTOR signaling pathway.

### Autophagy, apoptosis and G2/M phase arrest are mediated by the activation of ROS/JNK signaling and inhibition of the Akt/mTOR signaling pathway

To determine whether celastrol-induced autophagy, apoptosis and G2/M phase arrest are related to ROS/JNK signaling and the Akt/mTOR signaling pathway, NAC, SP, MK and Ra were used for further investigation. As shown in Fig. 7a, NAC and SP could reverse the cell death induced by celastrol as determined by the CCK8 analysis. However, in the presence of MK and Ra, cell viability was lower than that observed with the celastrol treatment alone. Flow cytometric analysis demonstrated that although both NAC and SP attenuated the celastrol-induced G2/M phase arrest, the effect of NAC was more obvious than that of SP. Compared with NAC and SP, MK and Ra had the opposite effect, leading to significantly increased G2/M phase arrest induced by celastrol (Fig. 7b). These results indicated that in the process of cell death and G2/M phase arrest, ROS/JNK signaling was activated and Akt/mTOR was suppressed by celastrol. The western blotting analysis showed that NAC and SP reduced the expression of apoptosis-related and autophagy-related proteins in the presence of celastrol. In contrast, MK and Ra increased the levels of apoptosis and autophagy-related proteins in the presence of celastrol. (Fig. 7c). In summary, celastrol induced G2/M phase arrest and triggered apoptosis and autophagy by activating ROS/JNK signalilng and blocking the Akt/mTOR signaling pathway.

### Celastrol inhibits the growth of gliomas in vivo

**Fig. 8:**
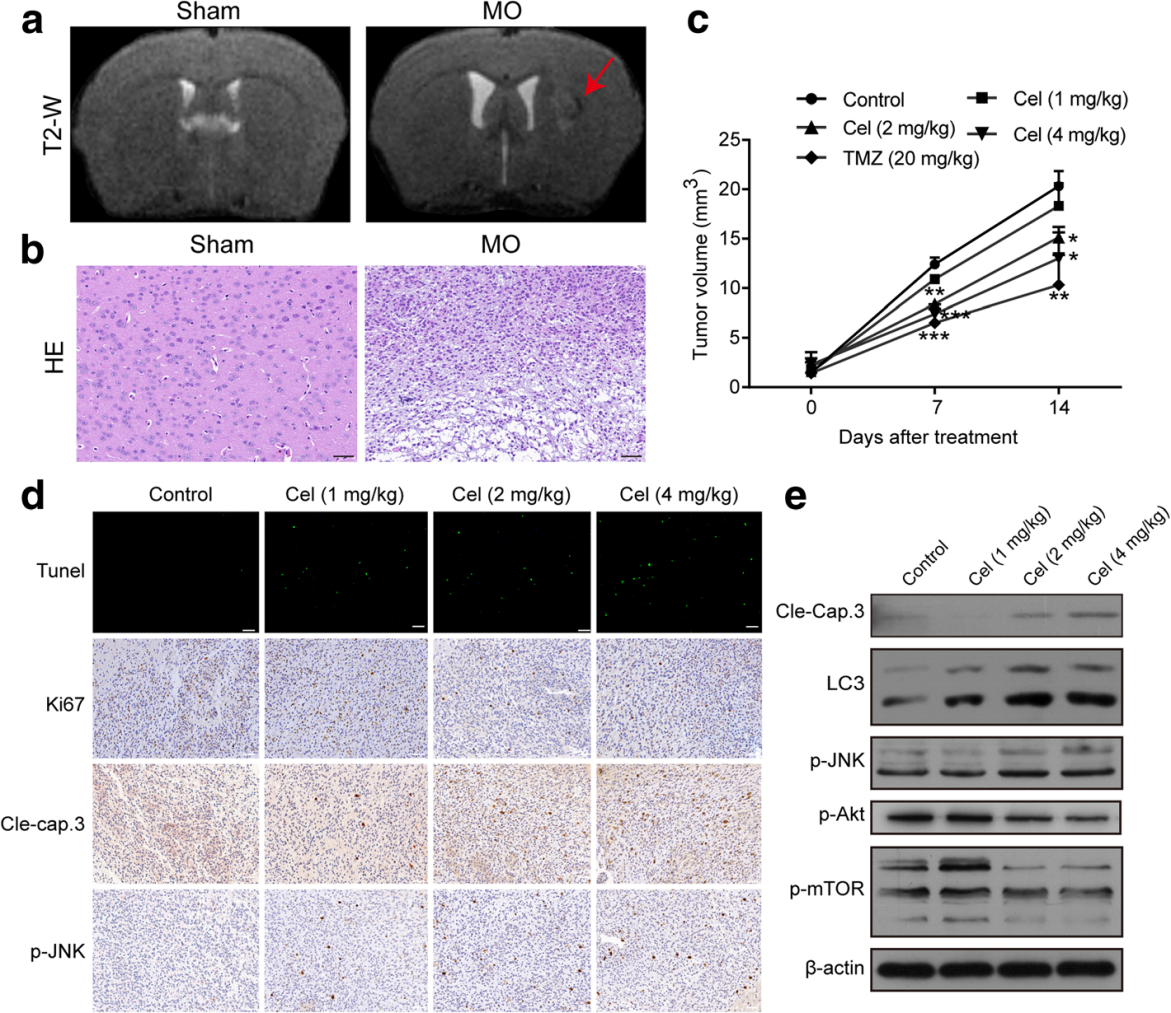
Celastrol suppressed the growth of orthotopic xenografts of U251 cells in vivo. **a** One week after inoculation, MRI scanning of glioma was performed on a 7.0 T MR scanner. **b** One week after inoculation, H&E staining of tumor tissue was carried out. Scale bars = 50 μm (**c)** Tumor volumes determined by MRI at 0, 7 and 14 days after inoculation in the 5 different groups. **d** Tunel assay and immunohistochemical staining of tumor specimens. Scale bars = 50 μm. **e** The levels of cleaved caspase-3, LC3B, p-JNK, p-Akt and p-mTOR in tumor tissues were detected by western blotting. β-actin was used as an internal control. Data are presented as the Mean ± SD (*n* = 3). *P < 0.05, **P < 0.01, ***P < 0.001, significantly different compared with the untreated control group

To evaluate the antitumor effect of celastrol in vivo, an orthotopic xenograft model of glioma was established by injecting U251 cells in the right striatum. Seven days after injection, T2-weighted MRI images of the brains of nude BALB/c mice were obtained. As shown in Fig. 8a, a distinct signal change occurred in the right caudate nucleus of the model group compared with the sham group. To further evaluate tumor formation, we performed hematoxylin and eosin staining of the brain. In the model group, we observed certain features of the tumor, such as significantly increased cell density, distinct nuclear atypia and common mitotic figures (Fig. 8b). These results suggested the success of model building. At this point, BALB/c nude mice were randomly assigned to the control, celastrol (1 mg/kg), celastrol (2 mg/kg), celastrol (4 mg/kg) or TMZ (20 mg/kg) groups. The mice in the treatment groups were injected with the corresponding medicine, while the mice in the control group were injected with PBS intraperitoneally every other day for a total of seven times. T2-weighted MRI images were obtained every 7 days. As shown in Fig. 8c, the celastrol groups at 2 and 4 mg/kg and the TMZ group showed inhibited tumor growth compared to the control group. H&E staining of important organs suggested that treatment with celastrol at 1, 2, and 4 mg/kg did not cause major organ-related toxicity (Additional file 6: Figure S6). Figure 8d, e and Additional file 7: Figure S7 show that celastrol increased the levels of cleaved caspase-3, LC3B and p-JNK and decreased the expression of p-Akt and mTOR. A TUNEL assay and immunohistochemistry demonstrated that celastrol-treated tumor tissues exhibited a significant increase in the level of TUNEL-positive cells and the levels of cleaved caspase-3 and JNK phosphorylation but decreased the level of Ki67 (Fig. 8d). Therefore, celastrol exhibited potent antitumor activity in vivo.

## Discussion

Glioma is a lethal human malignant tumor with the characteristics of high incidence, high recurrence rate, high mortality and limited treatment process [31]. Celastrol has attracted widespread concern, especially for its potent anti-tumor activity, including glioma [32–34]. Previous studies have shown that celastrol inhibited the growth of rat glioma cells and human glioma cells via the suppression of VEGFR expression and induction of apoptosis and cell cycle arrest [11, 35]. However, whether autophagy is involved in the inhibitory effect of celastrol on glioma cells is unclear and the mechanism of apoptosis has not been clarified. In the present study, our results further demonstrated that celastrol could inhibit the proliferation of glioma cells and cause G2/M phase arrest and apoptosis. More importantly, we found that celastrol simultaneously trigged apoptosis and autophagy by activating ROS/JNK signaling and suppressing the AKT/mTOR signaling pathway.

Recent studies have indicated that the heterogeneity of tumors is a major factor in determining cancer progression and the drug resistance response [36–38]. Tumor heterogeneity occurs not only between different individuals, but also between the same individuals because of spatial and temporal heterogeneity [39, 40]. Our results showed that celastrol exhibited different cytotoxicity in U251, U87-MG and C6 cells. U251 and C6 cells treated with celastrol for 24 h had a similar IC_50_ whereas those treated with celastrol for 48 h had significantly different IC_50_ values. In addition, U251 and U87 malignant glioma cell lines are derived from different individuals who suffered from a pleomorphic glioma. C6 glioma cell line is derived from N-nitrosomethylurea-induced rat glioma. Studies have shown that the U87 and U251 cell lines show differences in protein expression profiles [41]. These differences may also account for the different biological phenotypes such as proliferation, migration and invasion of glioma cell lines.

The G2/M cell cycle checkpoint plays a key role in normal cell proliferation. When DNA is damaged, Chk2 is firstly phosphorylated, and finally activation of Chk2 is achieved by autophosphorylation and transphosphorylation. Activation of Chk2 results in the phosphorylation of Cdc25, which leads to the inhibition of Cdc25. Cdc25 normally activates the cdc2/cyclin B1complex, which is a specific regulator of the G2/M phase [15, 42, 43]. Previous studies have reported celastrol induced cell cycle arrest at the G2/M phase in C6 cells [35]. According to the flow cytometry analysis, celastrol enhanced the proportion of glioma cells in the G2/M phase in all three glioma cell lines. However, in the U87-MG cells, celastrol also induced S phase arrest in the cell cycle. Similar conclusions have not been reported before. A further western blot analysis showed that celastrol upregulated the expression of the G2/M-phase-related proteins Chk2, p-Chk2, p-cdc2 and p-Cdc25C and downregulated the expression of Cdc25C. Surprisingly, celastrol increased the expression of cyclin B1 which was consistent with previous research [34]. Moreover, we found that celastrol also increased the protein level of p21, which plays a crucial role in blocking the activation of Cdk1/cyclin B1 in a p53-dependent or p53-independent manner [44]. Whether p53 participates in the inhibition of glioma cell proliferation induced by celastrol needs to be further tested.

Recent studies have extensively shown that two main apoptotic pathways participate in the regulation of apoptosis: extrinsic pathway and intrinsic pathway [45]. In the extrinsic apoptotic pathway, caspase-8 is first activated to cause subsequent downstream cascade reactions. The other pathway is the mitochondria-mediated intrinsic pathway, in which caspase-9 and additional caspase molecules such as caspase-3, -6 and -7 are activated [46]. Mitochondrial dysfunction has been shown to participate in the induction of apoptosis [47]. The present study found that a sharp decrease in MMP occurred after celastrol treatment. In normal cells, each caspase is present in an inactive state until it is cleaved after apoptotic signaling events [48]. Further western blotting analysis showed that treatment of celastrol led to the activation of caspase-3, caspase-8, caspase-9 and cleaved-PARP. Immunohistochemical and western blotting analyses confirmed that celastrol enhanced cleaved caspase-3 levels in vivo, and the TUNEL assay demonstrated a distinct increase in apoptosis in tumor tissues following celastrol treatment. In summary, celastrol can induce cell apoptosis in both the extrinsic and intrinsic pathways in glioma cells.

As a modulator of pathogenesis, autophagy has been widely studied as a promising, novel therapeutic target in diverse diseases [49]. In the present study, we found that celastrol triggered autophagy and elevated the expression of Beclin-1 and LC3B. Interestingly, the level of p62 also increased, which is related to the degradation of autophagy. When we used CQ (an autophagy inhibitor) to limit the degradation of the autophagosome, the expression of LC3B did not significantly change compared with that of the celastrol treatment alone. Moreover, the higher LC3B levels with celastrol + CQ than with CQ alone may indicate that celastrol increases the synthesis of autophagy-related membranes and partially blocks autophagic flux [50]. Meanwhile, the confocal results indicated that the number of yellow autophagic vesicles increased. Therefore, celastrol may partly inhibit the function of lysosomes. In addition, p62 has a critical role in oxidative stress response pathways [51]. The increased expression of p62 may also be associated with the generation of ROS. We further revealed that the autophagy inhibitors 3-MA and CQ moderately reinforced the inhibitory effect of celastrol on cell viability, indicating that celastrol-triggered autophagy may promote survival, which is consistent with the basic function of autophagy.

Autophagy and apoptosis can collectively trigger cell death through synergy, complementary cooperation, or alternative mechanisms [52]. In our study, suppression of autophagy following 3-MA and CQ treatment enhanced the level of apoptosis induced by celastrol. Moreover, when apoptosis was blocked by z-VAD, the expression of autophagy-related proteins was moderately increased. Therefore, the relationship of apoptosis and autophagy triggered by celastrol in glioma cells may be interdependent and interactive, which needs to be further confirmed. The pro-survival effect of autophagy caused by celastrol also indicated that autophagy and apoptosis caused by celastrol were antagonistic.

In glioma cells, anticancer agents generally induce an increase in ROS levels triggering the cell death [53]. However, tumor cells can survive under high levels of ROS by the nuclear factor erythroid 2-related factor 2 (Nrf2) pathway, a transcription gene regulator [54]. Moreover, ROS-dependent ERK activation has been shown to contribute to the invasion/migration of U87 glioma cells [55]. In the present study, celastrol induced a significant increase in ROS generation. The ROS inhibitor NAC markedly repressed the proliferation inhibition, cell cycle arrest, apoptosis and autophagy triggered by celastrol. All these results indicated that celastrol induced the generation of ROS, which contributed to cell death.

Usually, ROS is the upstream signal molecule of JNK, which can phosphorylate JNK and induce sustained JNK activation [56–58]. In addition, the activation of JNK promotes ROS production. A positive feedback effect or antagonistic effect may occur between ROS and JNK [34, 59]. Our study showed that NAC could slightly attenuate the phosphorylation of JNK while ROS generation was generally increased after pretreatment with SP in the cells treated with celastrol. An antagonistic effect may exist between ROS and JNK. Celastrol also upregulated the expression level of p38, another member of the MAPK family. Similar conclusions have been reported before [60]. The AKT/mTOR signaling pathway is one of the main growth regulatory pathways in both normal and cancer cells, and it can negatively regulate autophagy [61, 62]. Our study showed that celastrol inhibited Akt and mTOR phosphorylation and deactivated them. MK could slightly attenuate the phosphorylation of mTOR, while obvious changes in Akt phosphorylation were not observed after pretreatment with Ra in the cells treated with celastrol. Further, the present study suggested that the inhibition of ROS/JNK significantly inhibited autophagy and apoptosis, while the inhibition of Akt/mTOR had the opposite effect. Taken together, the above results suggested that celastrol triggered autophagy and apoptosis, which may be mediated through the activation of ROS/JNK signaling and inhibition of the Akt/mTOR signaling pathway.

Zhou and Huang et al. demonstrated that celastrol inhibited the growth of glioma in vitro and in vivo using murine ectopic xenografts models of glioma [10, 11]. Our in vivo data demonstrated that after celastrol treatment at doses of 2 and 4 mg/kg, significant antitumor effects were observed in the murine orthotopic transplantation model of glioma. Furthermore, the western blotting and immunohistochemical analyses showed significant increases in the level of cleaved caspase-3, LC3B and JNK phosphorylation but decreases in the level of Ki67, Akt and mTOR phosphorylation.

## Conclusions

**Fig. 9:**
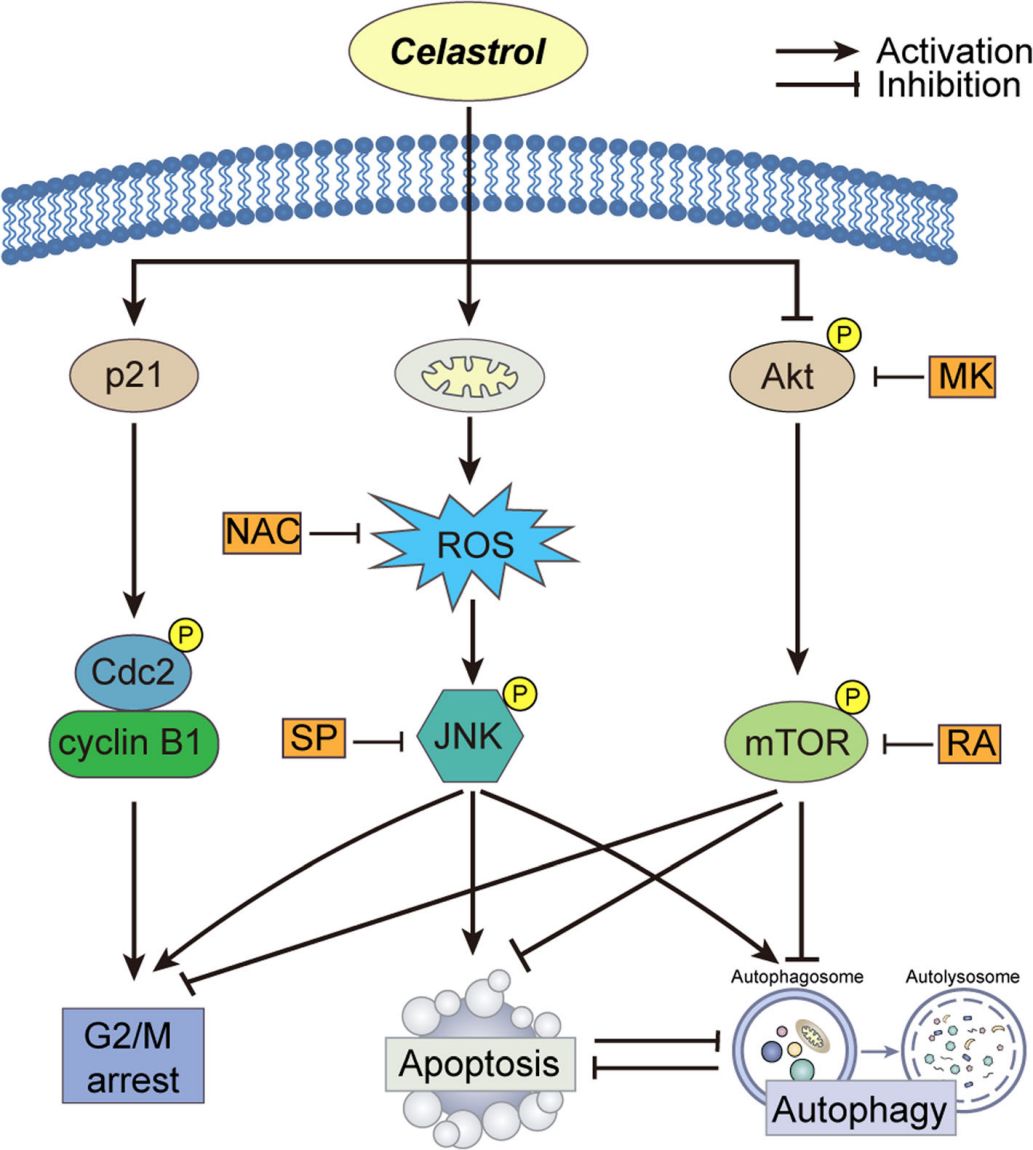
Proposed mechanism of celastrol-induced apoptosis and autophagy in glioma cells

In conclusion, our study showed that the antitumor effects of celastrol in glioma cells are related to G2/M phase arrest and the triggering of autophagy and apoptosis via the activation of ROS/JNK signaling and blocking of the Akt/mTOR signaling pathway. Further research indicated that autophagy induced by celastrol promoted glioma cell survival. Autophagy and apoptosis caused by celastrol in glioma cells may inhibit each other. The results of this study provide insights into the activities of celastrol and indicate its potential role in glioma therapy (Fig. 9).

## Additional files

Additional file 1: **Figure S1.** Celastrol inhibited the proliferation of glioma cells. a. Three glioma cell lines (U251, U87-MG and C6) were treated with celastrol (0, 0.3, 1, 3 and 10 μM) for 24 h. Cell morphology was observed using a Nikon microscope. Scale bars = 200 μm. b. U251, U87-MG and C6 cells were treated with celastrol (0, 0.03, 0.1, 0.3 and 1 μM) for 24 h and left untreated for approximately 10 days to allow the generation of colony. Cell colony formation was evaluated by a clone-formation assay. c. Astrocyte cells were treated with celastrol (0, 10, 20, 30, 40 and 50 μM) for 24 h. Cell viability was measured by CCK8 assay. Data are presented as the Mean ± SD (*n* = 3). *P < 0.05, **P < 0.01, ***P < *0.001*, significantly different compared with the untreated control group. (DOCX 684 kb)

Additional file 2: **Figure S2.** The percentage of the cell population at the G1, S, and G2/M phases is represented as the mean ± SD of three independent experiments. (DOCX 128 kb)

Additional file 3: **Figure S3.** Apoptotic nuclear morphological changes were evaluated by Hoechst 33342 staining and observed under a fluorescence microscope. The proportion of apoptotic cells was quantified. Red arrows indicate chromatin condensation and nuclear fragmentation. Scale bars = 100 μm. Data are presented as the Mean ± SD (*n* = 3). **P < 0.01, ***P < 0.001, significantly different compared with the untreated control group. (DOCX 820 kb)

Additional file 4: **Figure S4.** Figure S4 Celastrol triggered autophagy in U251cells. a. U251 cells were transiently transfected with the mRFP-EGFP-LC3B plasmid for 24 h and then treated with or without celastrol (1.5 μM) for 24 h. The images were examined using a confocal microscope. Scale bars = 25 μm. b. CQ (25 μM) was added to cells 2 h before celastrol treatment. Then, cells were treated with celastrol for 24 h. Quantitative results of autophagy-related proteins P62 and Beclin-1. ***P < 0.001, significantly different compared with the untreated control group. ^##^*P < 0.01*, significantly different compared with the celastrol treatment group. (DOCX 1273 kb)

Additional file 5: **Figure S5.** Cells were preincubated with z-VAD or 3-MA or CQ, and then treated with celastrol for 24 h. Quantitative results of autophagy-related proteins LC3B, P62 and apoptosis-related proteins cleaved caspase-3, caspase-8, caspase-9 and cleaved PARP. *P < 0.05, **P < 0.01, ***P < 0.001, significantly different compared with the untreated control group. ^#^*P < 0.05*, ^##^*P < 0.01*, ^###^*P < 0.001*, significantly different compared with the celastrol treatment group. (DOCX 287 kb)

Additional file 6: **Figure S6.** H&E staining of important organs. Scale bars = 20 μm. (DOCX 2087 kb)

Additional file 7: **Figure S7.** Western blot analysis of Cleaved caspase-3, LC3B, phospho-JNK, phospho-Akt and phospho-mTOR expression in tumor tissues. *P < 0.05, **P < 0.01, ***P < 0.001, significantly different compared with the untreated control group. Data are presented as the mean ± SD (n = 3) (DOCX 181 kb)

## Abbreviations

3-MA: 3-methyladenine
AKT: Protein kinase B
CCK8: Cell counting kit-8
CQ: Chloroquine diphosphate salt
DMSO: Dimethyl sulfoxide
ERK: Extracellular regulated protein kinases
FBS: Fetal bovine serum
FITC: Fluorescein isothiocyanate
GFP: Green fluorescent protein
JNK: c-Jun N-terminal kinase
MAPK: Mitogen-activated protein kinase
MEM: Minimum Eagle’s medium
MK: MK2206
MMP: Mitochondrial membrane potential
mTOR: Mammalian target of rapamycin
NAC: N-acetyl-L-cysteine
NEAA: Non-essential amino acids
PARP: Poly ADP-ribose polymerase
PBS: Phosphate buffer saline
PCD: Programmed cell death
PE: Phycoerythrin
PI: Propidiumiodide
Ra: Rapamycin
RFP: Red fluorescent protein
ROS: Reactive oxygen species
SP: SP600125
TMZ: Temozolomide
TUNEL: Terminal deoxynucleotidyl transferase -mediated dUTP Nick-End Labeling
z-VAD: z-VAD-fmk
