# Correction to: Celastrol mediates autophagy and apoptosis via the ROS/JNK and Akt/mTOR signaling pathways in glioma cells

**DOI:** 10.1186/s13046-019-1285-x

**Published:** 2019-07-02

**Authors:** Xihong Liu, Peiyuan Zhao, Xiujuan Wang, Lei Wang, Yingjun Zhu, Yadi Song, Wei Gao

**Affiliations:** 1 School of Traditional Chinese Medicine, Capital Medical University, Beijing, China; 2 Beijing Key Lab of TCM Collateral Disease Theory Research, Capital Medical University, Beijing, China; 3 Advanced Innovation Center for Human Brain Protection, Capital Medical University, Beijing, China; 4 Basic Discipline of Integrated Chinese and Western Medicine, Henan University of Chinese Medicine, Zhengzhou, Henan China

**Correction to: J Exp Clin Cancer Res (2019) 38:184**

**https://doi.org/10.1186/s13046-019-1173-4**

In the original publication of this article [1], there are two errors.

The first error is in author affiliation information. The corrected author affiliation information is as given hereafter:

Xihong Liu1, Peiyuan Zhao4, Xiujuan Wang1*, Lei Wang1, Yingjun Zhu1, Yadi Song1 and Wei Gao1,2,3*.

1 School of Traditional Chinese Medicine, Beijing Key Lab of TCM Collateral Disease Theory Research, Capital Medical University, Beijing, China.

2 School of Pharmaceutical Sciences, Capital Medical University, Beijing, China.

3 Advanced Innovation Center for Human Brain Protection, Capital Medical University, Beijing, China.

4 Basic Discipline of Integrated Chinese and Western Medicine, Henan University of Chinese Medicine, Henan, China.

The second error is in Fig. 8d (panel 2 of Tunel assay, group of 1 mg/kg Cel). The revised Fig. 8 which includes 8d has now been included in this correction.

**Fig. 8:**
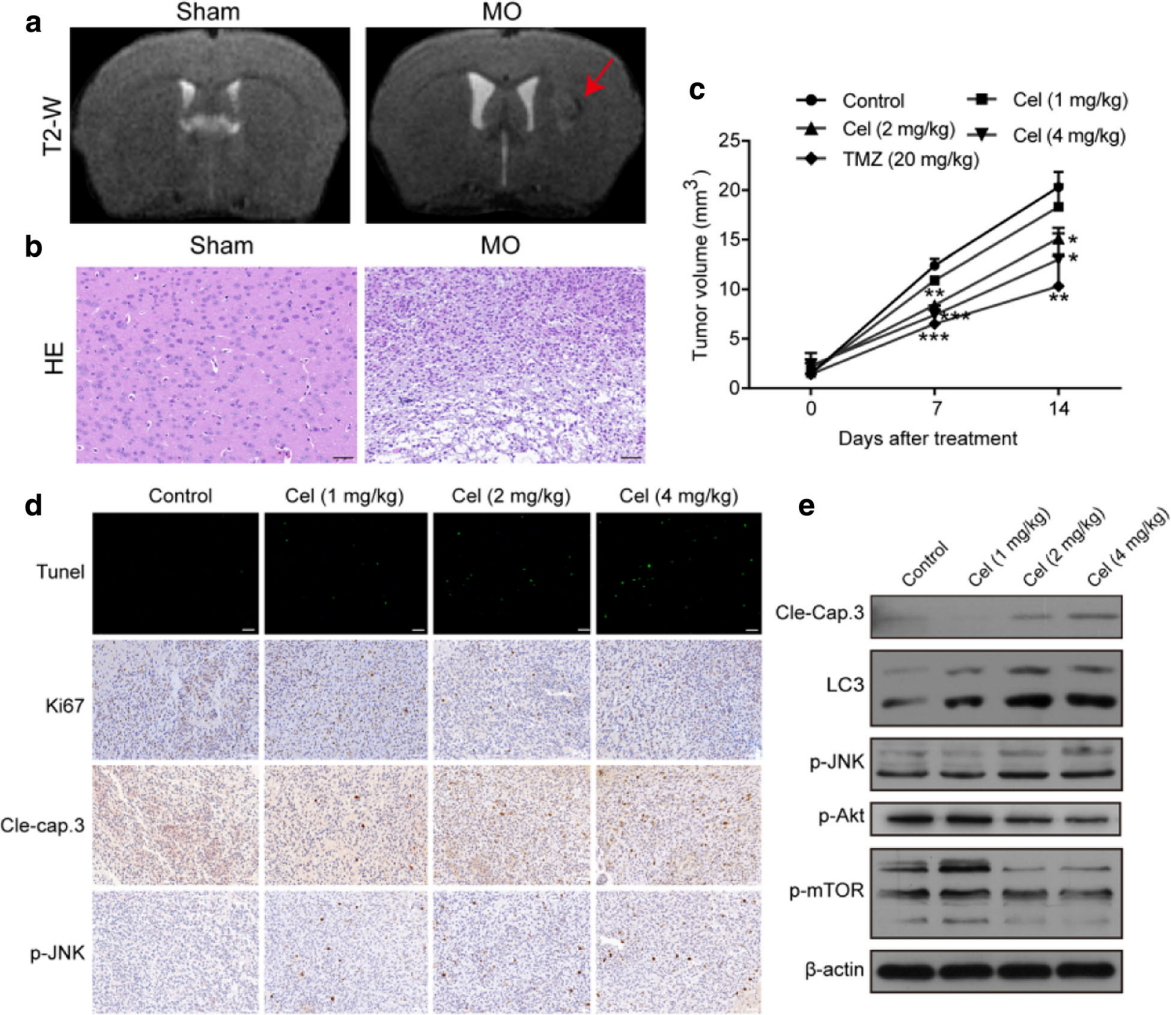
Celastrol suppressed the growth of orthotopic xenografts of U251 cells in vivo. **a** One week after inoculation, MRI scanning of glioma was performed on a 7.0 T MR scanner. **b** One week after inoculation, H&E staining of tumor tissue was carried out. Scale bars = 50 μm (**c**) Tumor volumes determined by MRI at 0, 7 and 14 days after inoculation in the 5 different groups. **d** Tunel assay and immunohistochemical staining of tumor specimens. Scale bars = 50 μm. **e** The levels of cleaved caspase-3, LC3B, p-JNK, p-Akt and p-mTOR in tumor tissues were detected by western blotting. β-actin was used as an internal control. Data are presented as the Mean ± SD (*n* = 3). *P < 0.05, **P < 0.01, ***P < 0.001, significantly different compared with the untreated control group

The correct Fig. 8 is given hereafter:

The error: In the initially published version of this article, the picture in the Tunel assay of Cel (1 kg/mg) group are the same with that in Cel (2 kg/mg) group in Fig. 8d.

This error does not affect discussions and conclusions drawn in the article.
