# Supplementary material for: Celastrol mediates autophagy and apoptosis via the ROS/JNK and Akt/mTOR signaling pathways in glioma cells
Source: J Exp Clin Cancer Res. 2019 May 3;38:184. doi: 10.1186/s13046-019-1173-4 (PMC6500040; doi:10.1186/s13046-019-1173-4)
Supplement: Supplementary file 1 — Figure S1. Celastrol inhibited the proliferation of glioma cells. a. Three glioma cell lines (U251, U87-MG and C6) were treated with celastrol (0, 0.3, 1, 3 and 10 μM) for 24 h. Cell morphology was observed using a Nikon microscope. Scale bars = 200 μm. b. U251, U87-MG and C6 cells were treated with celastrol (0, 0.03, 0.1, 0.3 and 1 μM) for 24 h and left untreated for approximately 10 days to allow the generation of colony. Cell colony formation was evaluated by a clone-formation assay. c. Astrocyte cells were treated with celastrol (0, 10, 20, 30, 40 and 50 μM) for 24 h. Cell viability was measured by CCK8 assay. Data are presented as the Mean ± SD (n = 3). *P < 0.05, **P < 0.01, ***P < 0.001, significantly different compared with the untreated control group. (DOCX 684 kb) [file 13046_2019_1173_MOESM1_ESM.docx]

**Fig. S1**


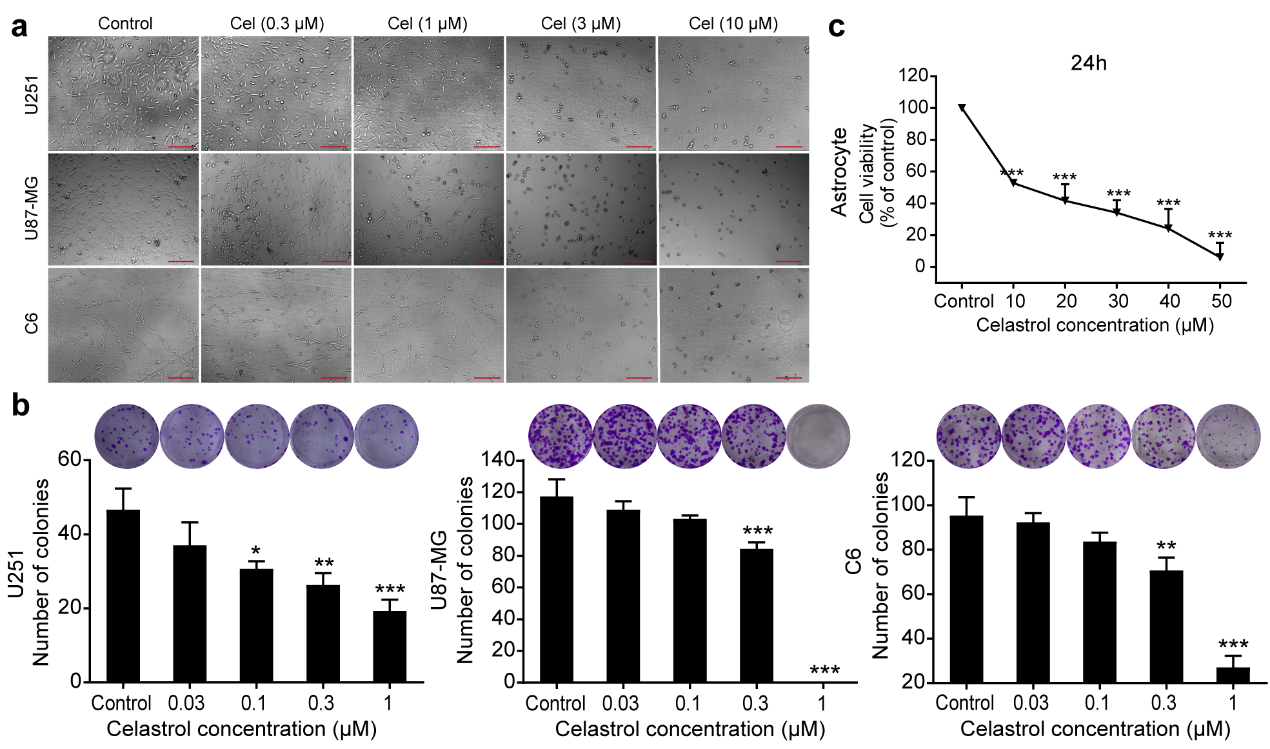


**Fig. S1** Celastrol inhibited the proliferation of glioma cells. a. Three glioma cell lines (U251, U87-MG and C6) were treated with celastrol (0, 0.3, 1, 3 and 10 μM) for 24h. Cell morphology was observed using a Nikon microscope. Scale bars=200 μm. b. U251, U87-MG and C6 cells were treated with celastrol (0, 0.03, 0.1, 0.3 and 1 μM) for 24 h and left untreated for approximately 10 days to allow the generation of colony. Cell colony formation was evaluated by a clone-formation assay. c. Astrocyte cells were treated with celastrol (0, 10, 20, 30, 40 and 50 μM) for 24h. Cell viability was measured by CCK-8 assay. Data are presented as the Mean ± SD (n=3). **P<0.05, **P<0.01, ***P<0.001*, significantly different compared with the untreated control group.
