# Supplementary material for: Celastrol mediates autophagy and apoptosis via the ROS/JNK and Akt/mTOR signaling pathways in glioma cells
Source: J Exp Clin Cancer Res. 2019 May 3;38:184. doi: 10.1186/s13046-019-1173-4 (PMC6500040; doi:10.1186/s13046-019-1173-4)
Supplement: Supplementary file 2 — Figure S2. The percentage of the cell population at the G1, S, and G2/M phases is represented as the mean ± SD of three independent experiments. (DOCX 128 kb) [file 13046_2019_1173_MOESM2_ESM.docx]

**Fig. S2**


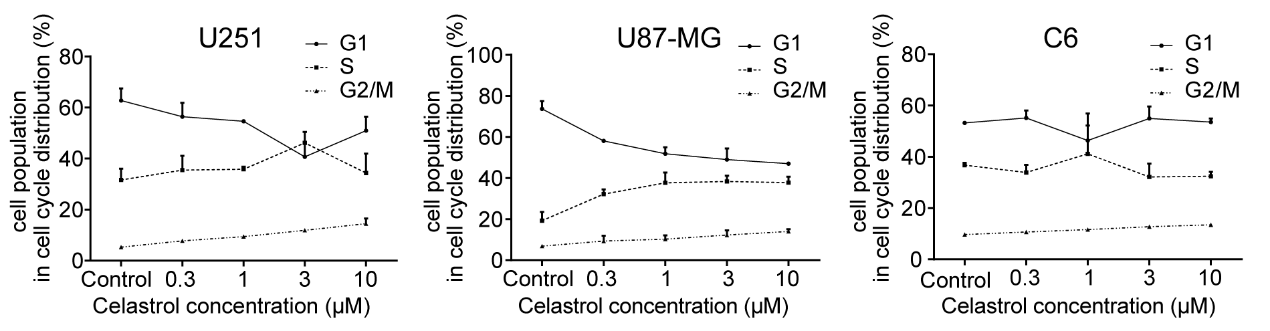


**Fig. S2** The percentage of the cell population at the G1, S, and G2/M phases is represented as the mean ± SD of three independent experiments.
