# Supplementary material for: Celastrol mediates autophagy and apoptosis via the ROS/JNK and Akt/mTOR signaling pathways in glioma cells
Source: J Exp Clin Cancer Res. 2019 May 3;38:184. doi: 10.1186/s13046-019-1173-4 (PMC6500040; doi:10.1186/s13046-019-1173-4)
Supplement: Supplementary file 3 — Figure S3. Apoptotic nuclear morphological changes were evaluated by Hoechst 33342 staining and observed under a fluorescence microscope. The proportion of apoptotic cells was quantified. Red arrows indicate chromatin condensation and nuclear fragmentation. Scale bars = 100 μm. Data are presented as the Mean ± SD (n = 3). **P < 0.01, ***P < 0.001, significantly different compared with the untreated control group. (DOCX 820 kb) [file 13046_2019_1173_MOESM3_ESM.docx]

**Fig. S3**


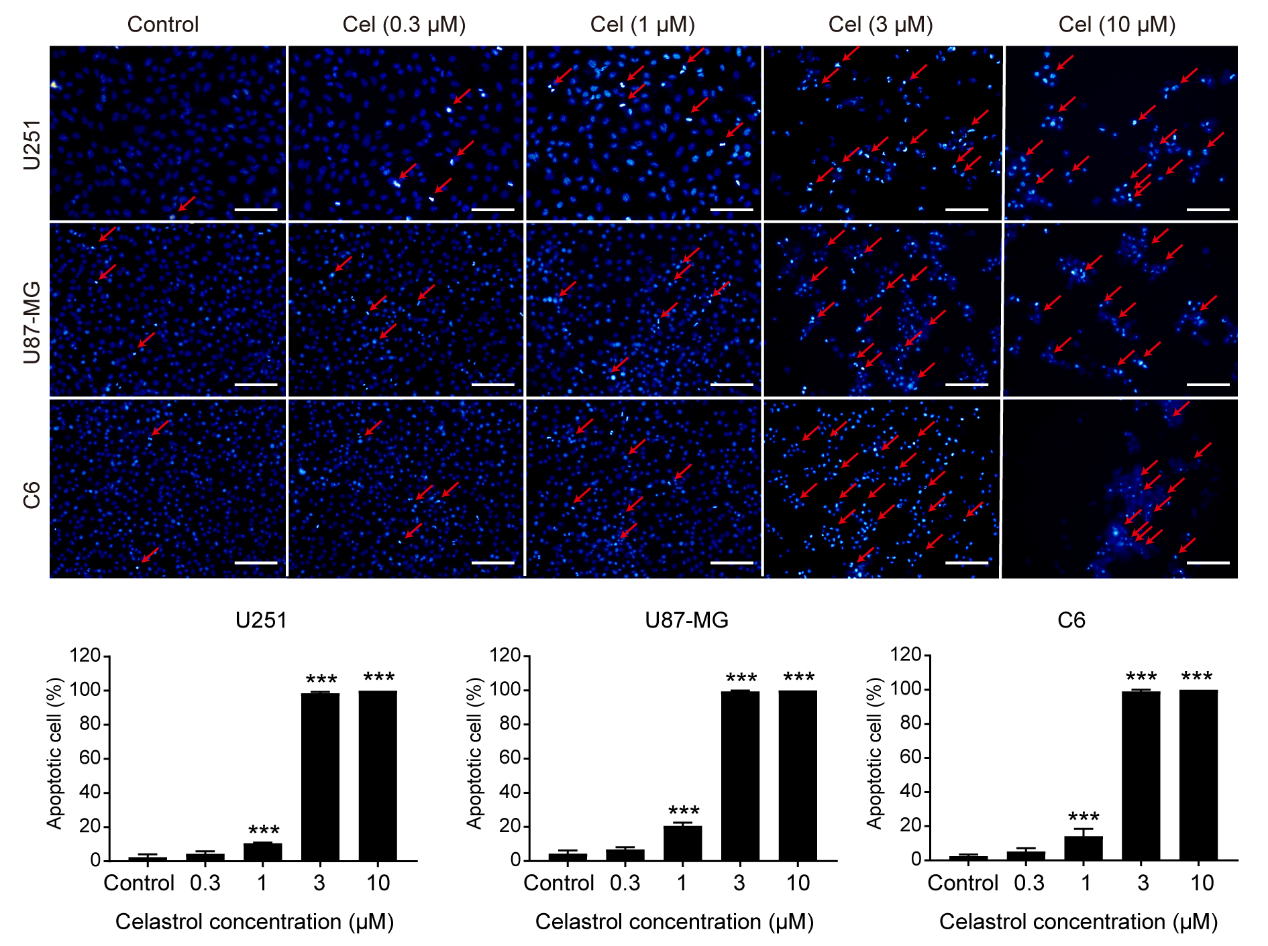


**Fig. S3** Apoptotic nuclear morphological changes were evaluated by Hoechst 33342 staining and observed under a fluorescence microscope. The proportion of apoptotic cells was quantified. Red arrows indicate chromatin condensation and nuclear fragmentation. Scale bars=100 μm. Data are presented as the Mean ± SD (n=3). ****P<0.001*, significantly different compared with the untreated control group.
