# Supplementary material for: Celastrol mediates autophagy and apoptosis via the ROS/JNK and Akt/mTOR signaling pathways in glioma cells
Source: J Exp Clin Cancer Res. 2019 May 3;38:184. doi: 10.1186/s13046-019-1173-4 (PMC6500040; doi:10.1186/s13046-019-1173-4)
Supplement: Supplementary file 4 — Figure S4. Figure S4 Celastrol triggered autophagy in U251cells. a. U251 cells were transiently transfected with the mRFP-EGFP-LC3B plasmid for 24 h and then treated with or without celastrol (1.5 μM) for 24 h. The images were examined using a confocal microscope. Scale bars = 25 μm. b. CQ (25 μM) was added to cells 2 h before celastrol treatment. Then, cells were treated with celastrol for 24 h. Quantitative results of autophagy-related proteins P62 and Beclin-1. ***P < 0.001, significantly different compared with the untreated control group. ##P < 0.01, significantly different compared with the celastrol treatment group. (DOCX 1273 kb) [file 13046_2019_1173_MOESM4_ESM.docx]

**Fig. S4**


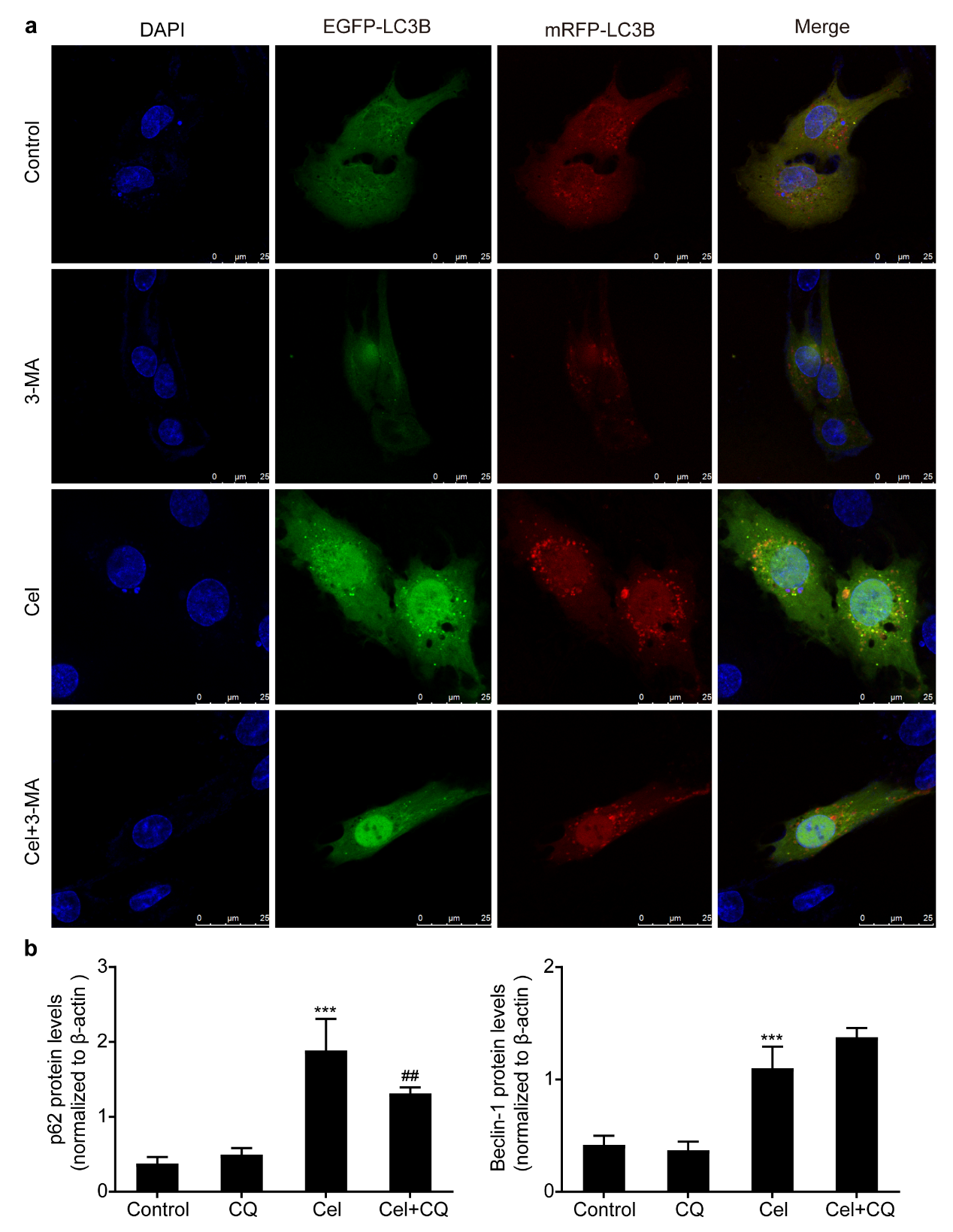


**Fig. S4** Celastrol triggered autophagy in U251cells. a. U251 cells were transiently transfected with the mRFP-EGFP-LC3B plasmid for 24 h and then treated with or without celastrol (1.5 μM) for 24 h. The images were examined using a confocal microscope. Scale bars=25 μm. b. CQ (25 μM) was added to cells 2 h before celastrol treatment. Then, cells were treated with celastrol for 24 h. Quantitative results of autophagy-related proteins P62 and Beclin-1. ****P<0.001*, significantly different compared with the untreated control group. ^##^*P<0.01*, significantly different compared with the celastrol treatment group.
