# Supplementary material for: Celastrol mediates autophagy and apoptosis via the ROS/JNK and Akt/mTOR signaling pathways in glioma cells
Source: J Exp Clin Cancer Res. 2019 May 3;38:184. doi: 10.1186/s13046-019-1173-4 (PMC6500040; doi:10.1186/s13046-019-1173-4)
Supplement: Supplementary file 5 — Figure S5. Cells were preincubated with z-VAD or 3-MA or CQ, and then treated with celastrol for 24 h. Quantitative results of autophagy-related proteins LC3B, P62 and apoptosis-related proteins cleaved caspase-3, caspase-8, caspase-9 and cleaved PARP. *P < 0.05, **P < 0.01, ***P < 0.001, significantly different compared with the untreated control group. #P < 0.05, ##P < 0.01, ###P < 0.001, significantly different compared with the celastrol treatment group. (DOCX 287 kb) [file 13046_2019_1173_MOESM5_ESM.docx]

**Fig. S5**

**
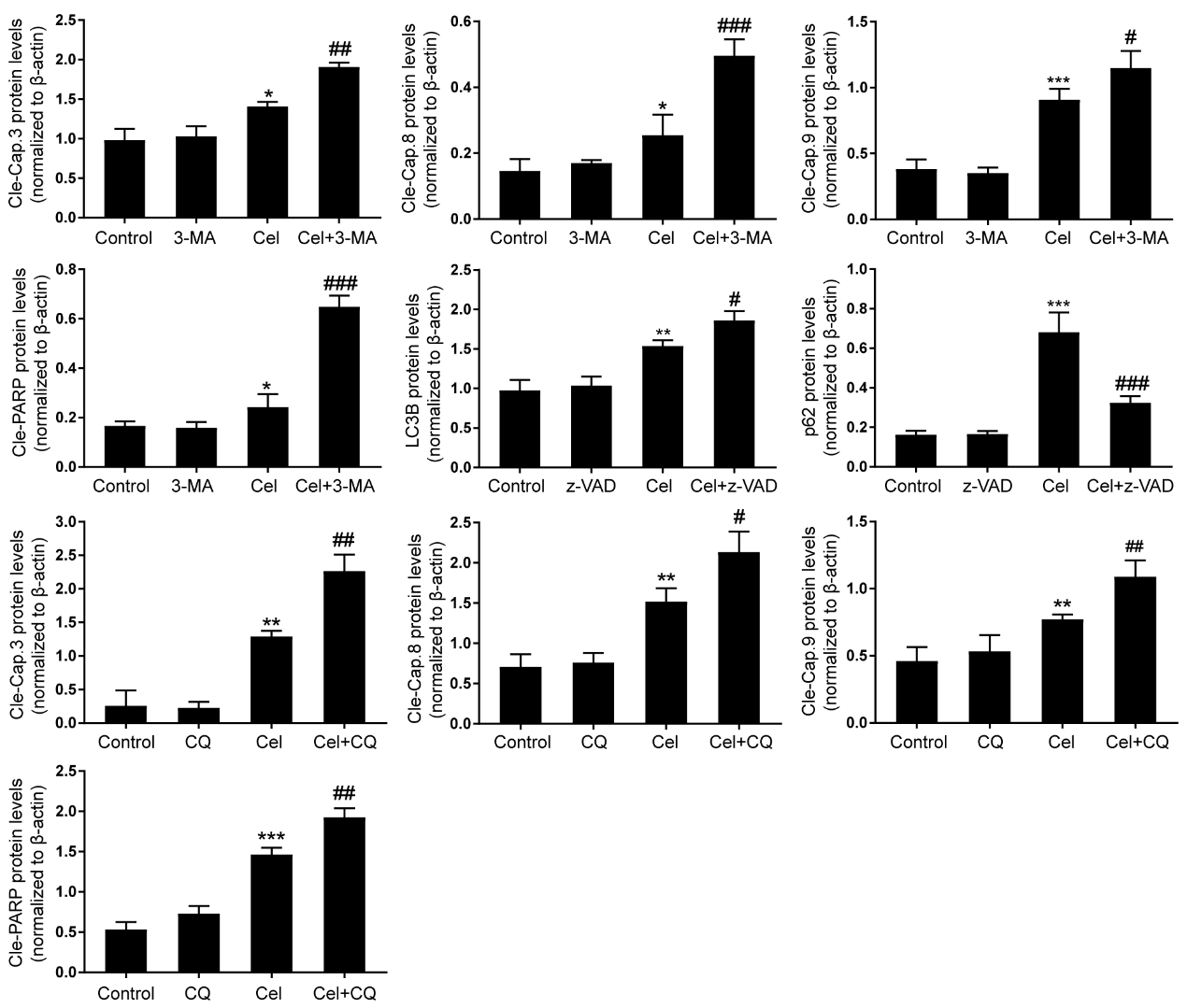
**

**Fig. S5** Cells were preincubated with z-VAD or 3-MA or CQ, and then treated with celastrol for 24 h. Quantitative results of autophagy-related proteins LC3B, P62 and apoptosis-related proteins cleaved caspase 3, caspase 8, caspase 9 and cleaved PARP. **P<0.05*, ***P<0.01*, ****P<0.001*, significantly different compared with the untreated control group. ^#^*P<0.05*, ^##^*P<0.01*, ^###^*P<0.001*, significantly different compared with the celastrol treatment group.
