# Supplementary figures and images for: Celastrol mediates autophagy and apoptosis via the ROS/JNK and Akt/mTOR signaling pathways in glioma cells
Source: J Exp Clin Cancer Res. 2019 May 3;38:184. doi: 10.1186/s13046-019-1173-4 (PMC6500040; doi:10.1186/s13046-019-1173-4)

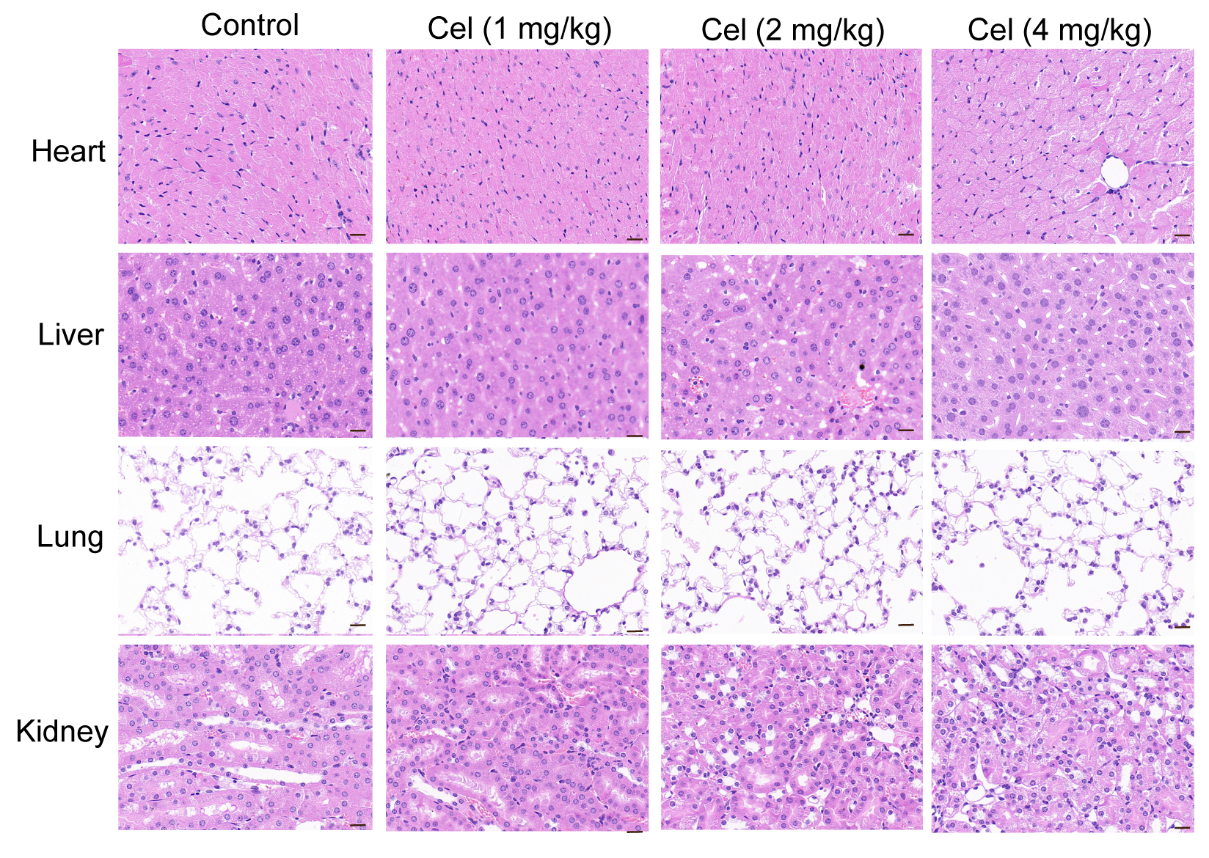


**Fig. S6** H&E staining of important organs. Scale bars=20 μm.

Supplement: Supplementary file 6 — Figure S6. H&E staining of important organs. Scale bars = 20 μm. (DOCX 2087 kb) [file 13046_2019_1173_MOESM6_ESM.docx]
