# Supplementary material for: Celastrol mediates autophagy and apoptosis via the ROS/JNK and Akt/mTOR signaling pathways in glioma cells
Source: J Exp Clin Cancer Res. 2019 May 3;38:184. doi: 10.1186/s13046-019-1173-4 (PMC6500040; doi:10.1186/s13046-019-1173-4)
Supplement: Supplementary file 7 — Figure S7. Western blot analysis of Cleaved caspase-3, LC3B, phospho-JNK, phospho-Akt and phospho-mTOR expression in tumor tissues. *P < 0.05, **P < 0.01, ***P < 0.001, significantly different compared with the untreated control group. Data are presented as the mean ± SD (n = 3) (DOCX 181 kb) [file 13046_2019_1173_MOESM7_ESM.docx]

**Fig. S7**


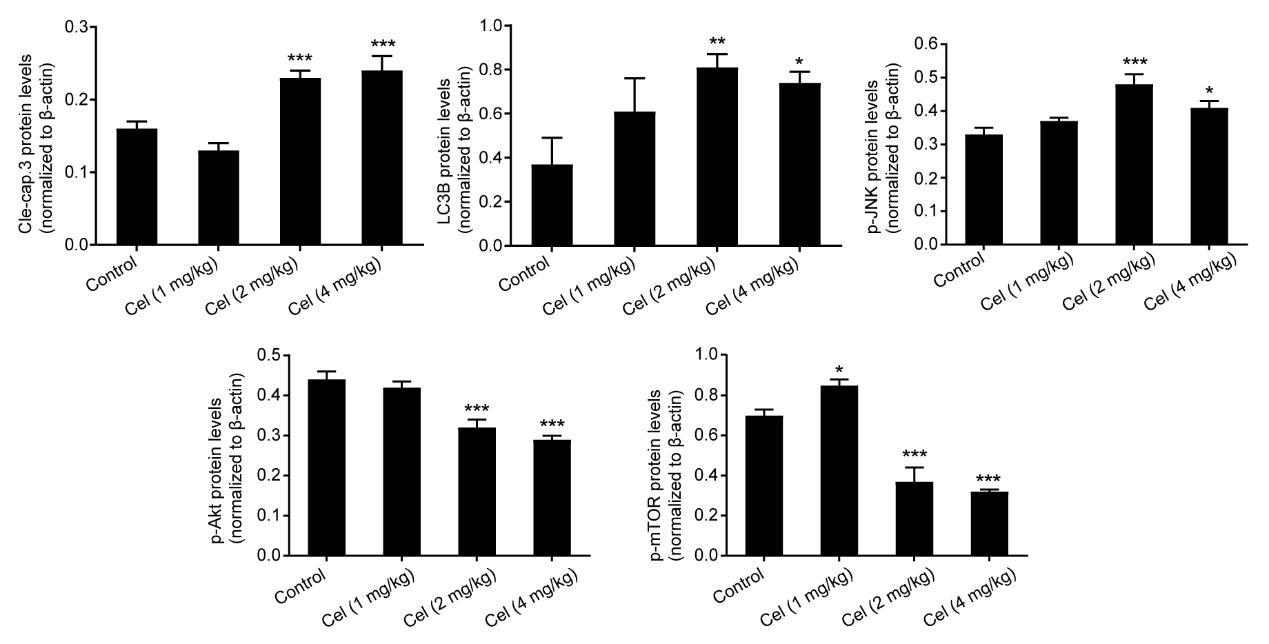


**Fig. S7** Western blot analysis of cleaved caspase-3, LC3B, phospho-JNK, phospho-Akt and phospho-mTOR expression in tumor tissues. **P*<0.05, ***P*<0.01, ****P*<0.001, significantly different compared with the untreated control group. Data are presented as the mean ± SD (n=3).
